# The co-evolution of the genome and epigenome in colorectal cancer

**DOI:** 10.1038/s41586-022-05202-1

**Published:** 2022-10-26

**Authors:** Timon Heide, Jacob Househam, George D. Cresswell, Inmaculada Spiteri, Claire Lynn, Maximilian Mossner, Chris Kimberley, Javier Fernandez-Mateos, Bingjie Chen, Luis Zapata, Chela James, Iros Barozzi, Ketevan Chkhaidze, Daniel Nichol, Vinaya Gunasri, Alison Berner, Melissa Schmidt, Eszter Lakatos, Ann-Marie Baker, Helena Costa, Miriam Mitchinson, Rocco Piazza, Marnix Jansen, Giulio Caravagna, Daniele Ramazzotti, Darryl Shibata, John Bridgewater, Manuel Rodriguez-Justo, Luca Magnani, Trevor A. Graham, Andrea Sottoriva

**Affiliations:** 1 Centre for Evolution and Cancer, The Institute of Cancer Research, London, UK; 2 Computational Biology Research Centre, Human Technopole, Milan, Italy; 3 Evolution and Cancer Lab, Centre for Genomics and Computational Biology, Barts Cancer Institute, Queen Mary University of London, London, UK; 4 Department of Surgery and Cancer, Imperial College London, London, UK; 5 Centre for Cancer Research, Medical University of Vienna, Vienna, Austria; 6 Department of Pathology, UCL Cancer Institute, University College London, London, UK; 7 Department of Medicine and Surgery, University of Milano-Bicocca, Milan, Italy; 8 Department of Mathematics and Geosciences, University of Triest, Triest, Italy; 9 Department of Pathology, University of Southern California Keck School of Medicine, Los Angeles, CA USA; 10 UCL Cancer Institute, University College London, London, UK

**Keywords:** Cancer genomics, Epigenomics, Tumour heterogeneity, Computational biology and bioinformatics, Colorectal cancer

## Abstract

Colorectal malignancies are a leading cause of cancer-related death^1^ and have undergone extensive genomic study^2,3^. However, DNA mutations alone do not fully explain malignant transformation^4–7^. Here we investigate the co-evolution of the genome and epigenome of colorectal tumours at single-clone resolution using spatial multi-omic profiling of individual glands. We collected 1,370 samples from 30 primary cancers and 8 concomitant adenomas and generated 1,207 chromatin accessibility profiles, 527 whole genomes and 297 whole transcriptomes. We found positive selection for DNA mutations in chromatin modifier genes and recurrent somatic chromatin accessibility alterations, including in regulatory regions of cancer driver genes that were otherwise devoid of genetic mutations. Genome-wide alterations in accessibility for transcription factor binding involved CTCF, downregulation of interferon and increased accessibility for SOX and HOX transcription factor families, suggesting the involvement of developmental genes during tumourigenesis. Somatic chromatin accessibility alterations were heritable and distinguished adenomas from cancers. Mutational signature analysis showed that the epigenome in turn influences the accumulation of DNA mutations. This study provides a map of genetic and epigenetic tumour heterogeneity, with fundamental implications for understanding colorectal cancer biology.

## Abstract (web summary)

A study maps genetic and epigenetic heterogeneity of primary colorectal adenomas and cancers at single-clone resolution through spatial multi-omic profiling of individual glands and adjacent normal tissue.

## Main

Clonal evolution, fuelled by intra-tumour heterogeneity, drives tumour initiation, progression and treatment resistance^8,9^. Much is known about the genetic evolution and intra-tumour heterogeneity of colorectal malignancies^2,3,10^. Although genetic heterogeneity is widespread^11^, epigenetic changes are also responsible for phenotypic variation between cancer cells^4–7^. Epigenetic profiling of chromatin accessibility in colon cancer has been performed in seminal studies in cell lines^12^ and human samples^13,14^. However, current investigations are limited to single-bulk samples and some also lack normal controls^14^. Moreover, how cancer genomes and epigenomes concomitantly evolve and shape intra-tumour genetic and epigenetic heterogeneity remains unexplored.

Measuring genome–epigenome co-evolution in a quantitative manner is possible by multi-omic profiling at single-clone resolution and accurate spatial sampling of human neoplasms, as well as matched normal tissue. Colorectal cancers (CRCs) are organized into glandular structures, reminiscent of the crypts in the normal intestinal epithelium^15^. Normal crypts are tube-like invaginations where cell proliferation is driven by a relatively small number of stem cells at the base^16–19^, and cancer glands are thought to have the same architecture^20^. This implies that all cells within a gland share a recent common ancestor and are a few cell divisions apart: thus, glands are largely clonal populations that, through cell proliferation, copy DNA with relatively high fidelity. Ultimately, the gland can be thought of as a natural whole-genome amplification machine that can be exploited to perform multi-omics at single-clone resolution. Indeed, single-crypt and single-gland genomic profiling has long been used to study clonal dynamics in both normal^21–23^ and cancer cells^10,24–29^. We developed a method to concomitantly profile single nucleotide variants (SNVs), copy-number alterations (CNAs), chromatin accessibility with transposase-accessible chromatin sequencing (ATAC-seq)^30^ and full transcriptomes with RNA sequencing (RNA-seq) from the same individual gland or crypt. Here we present the results of multi-region single-gland multi-omics of 1,370 samples from 38 lesions arising in 30 patients, with 21–55 tumour samples per patient (median = 42).

## Single-gland multi-omics

**Fig. 1:**
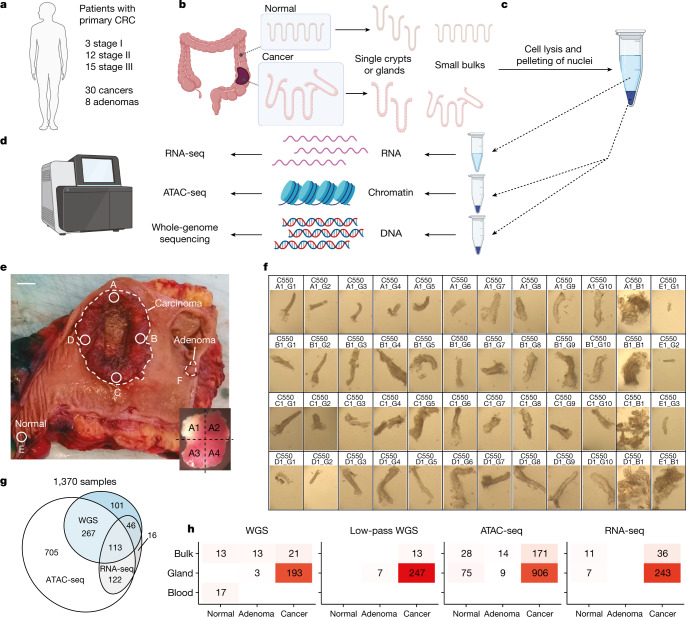
Spatial single-gland multi-omics. **a**, Fresh colectomy specimens from 30 patients with stage I–III CRC were used to collect tissue from 30 cancers and 8 adenomas. **b**, Single glands and small bulks (minibulks) were isolated from normal and neoplastic samples. **c**, We performed cell lysis followed by nuclei pelleting on each sample. **d**, Cytosolic fractions were used for RNA-seq whereas nuclei were used for WGS and ATAC-seq. **e**, We identified separate regions of the specimen: carcinoma (A, B, C and D), a distant normal sample (E) and adenomas if present (F, G and H). Each sample was split into 4 fragments (inset). Scale bar, 1 cm. **f**, From each fragment, we collected individual glands (labelled as _G) as well as minibulks (agglomerates of a few dozen crypts, labelled as _B). **g**, We performed multi-omics using WGS, ATAC-seq and RNA-seq on the same sample, achieving a good level of overlap between assays. **h**, For each assay, we had representative samples from normal, adenoma and cancer regions. Graphics in **b**–**d** were created with BioRender.com.

We prospectively collected fresh resection specimens from 30 stage I–III primary CRCs and 8 concomitant adenomas from 30 patients referred for surgery at the University College London Hospital (Fig. 1a, Methods, Supplementary Fig. 1 and Supplementary Table 1 for clinical information). Single-gland isolation was performed on normal and neoplastic tissue (Fig. 1b and Methods), followed by separation of nuclei from cytosol (Fig. 1c). Leftover fragments that remained after gland isolation were retained to assess how representative glands are of the bulk they originated from. We will refer to those samples, consisting of a few tens of glands, as minibulks. We used the nuclei to perform whole-genome sequencing (WGS) and chromatin accessibility profiling with ATAC-seq, and the cytosol to perform full transcriptome RNA-seq (Fig. 1d and Methods). We verified that cytosolic RNA expression in our normal colon tissue controls was highly correlated with whole-cell RNA expression from the The Cancer Genome Atlas cohort^2^ (Supplementary Fig. 2).

Our strategy of spatially sampling tumour tissue was designed to measure clonal evolution at multiple scales. We first sampled four spatially distant regions of a given cancer (regions A, B, C and D) located close to the tumour edge, one distant region of normal epithelium (region E) and concomitant adenomas if present (regions F, G and H). A bulk sample was collected from each region and was spatially annotated in the original resection specimen (Fig. 1e and Supplementary Fig. 1). Each piece was cut into four subregions (for example, A1–A4 and B1–B4) as shown in the inset of Fig. 1e. We then collected and profiled 12–40 (median = 37) individual glands and 2–17 (median = 4) minibulks from the tumours of each patient (Fig. 1f and additional figures at 10.6084/m9.figshare.19848199). Blood or, when unavailable, large adjacent normal tissue samples were used as normal reference.

ATAC-seq was performed in 18–59 samples per patient (median = 42; Methods and Supplementary Table 2), deep WGS (median depth 35×) was performed in 3–15 samples per patient (median = 8), and low-pass WGS (median depth 1.2×) was performed in 1–22 samples per patient (median = 8; Methods and Supplementary Table 3). For a proportion of tumour samples (*n* = 370/1,370), both WGS and ATAC-seq data were available (Fig. 1g). We also generated a total of 600 whole transcriptomes, of which 297 were of sufficient quality to be used for analysis (1–40 samples in 27 patients, median = 7; Methods and Supplementary Table 4) with many also overlapping the WGS dataset, the ATAC-seq dataset or both (Fig. 1h). In addition, we ran methylation arrays on 8 samples (Methods). We identified CNAs, somatic SNVs, short insertions and deletions (indels) and ATAC-seq peaks for all samples (Methods).

## Somatic mutations affecting the epigenome

**Fig. 2:**
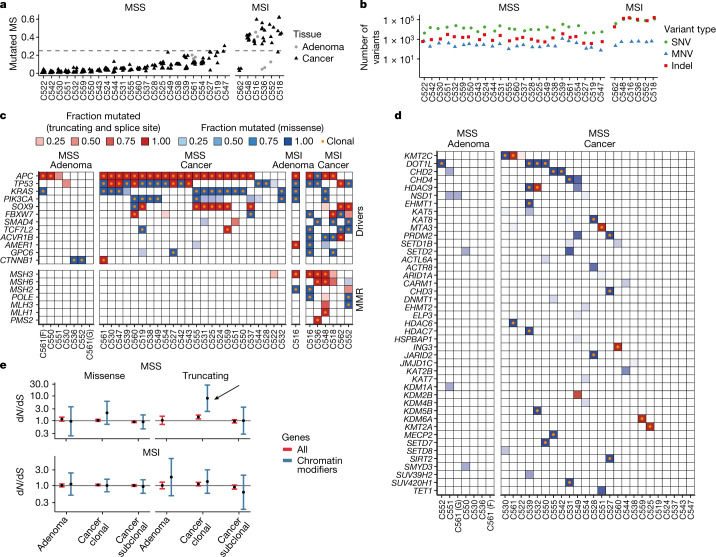
DNA alterations in canonical cancer drivers and chromatin modifier genes. **a**, MSI frequency per case. Each data point shows the fraction of mutated microsatellites reported by MSIsensor in a sample. More than 25% mutated microsatellites suggest MSI. **b**, Mutational burden by type of mutation across all cancer samples of a given case (MNV, multiple-nucleotide variant). **c**, Fraction of samples in which recurrently mutated CRC driver genes were mutated (shading) and the type of the corresponding mutation (colour). Orange dots indicate that the mutation is clonal (that is, present in all samples). MMR, mismatch repair. **d**, Truncating mutations and indels in chromatin modifier genes in MSS cases. **e**, d*n*/d*S* analysis of clonal and subclonal chromatin modifier mutations in MSS and MSI cancers and adenomas reveals significant selection in clonal truncating mutations in chromatin modifier genes in MSS carcinomas (see arrow). The error bars are 95% confidence interval; points show the maximum-likelihood estimate; numbers of cases were *n* = 7 and *n* = 24 for the MSS adenomas and cancers, respectively, and *n* = 1 and *n* = 6 for the MSI adenoma and cancers, respectively.

**Extended Data Fig. 1:**
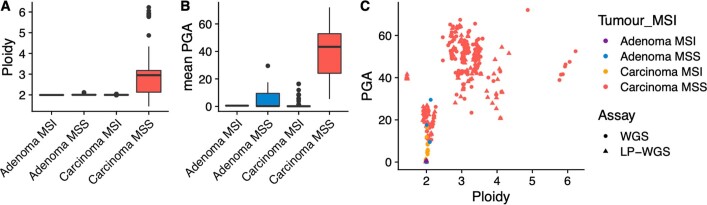
Chromosomal differences between adenomas and carcinomas. (A) Ploidy and (B) PGA (Percentage Genome Altered) of adenomas vs carcinomas, separated by MSI/MSS status (Number of samples per group: 10 Adenoma MSI, 13 Adenoma MSS, 66 Carcinoma MSI, 408 Carcinoma MSS). The lower and upper hinges of the boxes show the first and third quartiles, the black the horizontal lines show the medians. Whiskers extend to the most extreme values up to 1.5 inter quartile ranges from the whiskers and values outside of this range are shown as individual points. (C) Comparison of the two values.

We first assessed the landscape of genetic alterations in our cohort. Six cases were characterized by microsatellite instability (MSI; Methods), as reported in Fig. 2a, leading to substantially higher SNV and indel burdens (Fig. 2b). CNAs recapitulated previous datasets^2,3^, with microsatellite stable (MSS) cases exhibiting high aneuploidy and MSI cases being largely diploid (Supplementary Fig. 3). As previously described^3^, adenoma samples showed a lower degree of aneuploidy than MSS carcinomas, except for two outliers (Extended Data Fig. 1). Recurrent copy loss of canonical tumour suppressor genes, such as *APC*, *PTEN*, *TP53* and *SMAD4*, was confirmed. Focal amplifications were found in *FGFR1* (two cases) and *MYC* (one case). Recurrent cancer driver mutation events in CRCs were recapitulated in this dataset, with stereotypical mutations in *APC*, *KRAS* and *TP53* (Fig. 2c and additional figures at 10.6084/m9.figshare.19849138). Except for a two cases (C522 and C539), mutations in these three genes were invariably clonal. The mutational profiles of the adenomas were consistent with an earlier study^31^ for both *APC* (4/8 versus 73/135, *P* value = 1, Fisher’s exact test) and *KRAS* (2/8 versus 13/135, *P* value = 0.20, Fisher’s exact test) mutation frequencies. We observed a slightly larger incidence of *TP53* mutations in our study (2/8 versus 4/135, *P* value = 0.037, Fisher’s exact test). Adenoma mutation frequencies were similar to another previous study^3^ (*TP53*, *P* value = 1; *KRAS*, *P* value = 0.33; *APC*, *P* value = 0.029; *PIK3CA*, *P* value = 1; Fisher’s exact test).

To investigate the influence of genetic mutations on the epigenome, we examined somatic mutations in chromatin modifier genes (Supplementary Table 5), such as members of the lysine demethylase (KDM), lysine acetyltransferase (KAT), lysine methyltransferase (KMT) and SWI/SNF (ARID1A) families (see Fig. 2d for MSS cases, and Supplementary Fig. 4 for all). Evolutionary selection on chromatin modifier genes was assessed by d*n*/dS (refs. ^32,33^ and Methods). Clonal truncating mutations (occurring in all samples of a tumour) in chromatin modifier genes of MSS cases showed clear signs of positive selection, with d*n*/d*S* significantly >1 (Fig. 2e, arrow). Subclonal chromatin modifier mutations were present, but positive selection was not detected, with d*n*/d*S* ≈ 1 (Fig. 2e). No evidence of positive selection for chromatin modifier gene mutations was detected in MSI cancers, although their high mutational burden may limit the power of detection. Overall, clonal truncating mutations in chromatin modifiers were found in 6/24 MSS cases (25%) and all MSI cases, with few recurrently mutated genes, suggesting a convergent pattern of selection for inactivation of chromatin modifiers in CRC.

## Recurrent chromatin changes are largely clonal

**Fig. 3:**
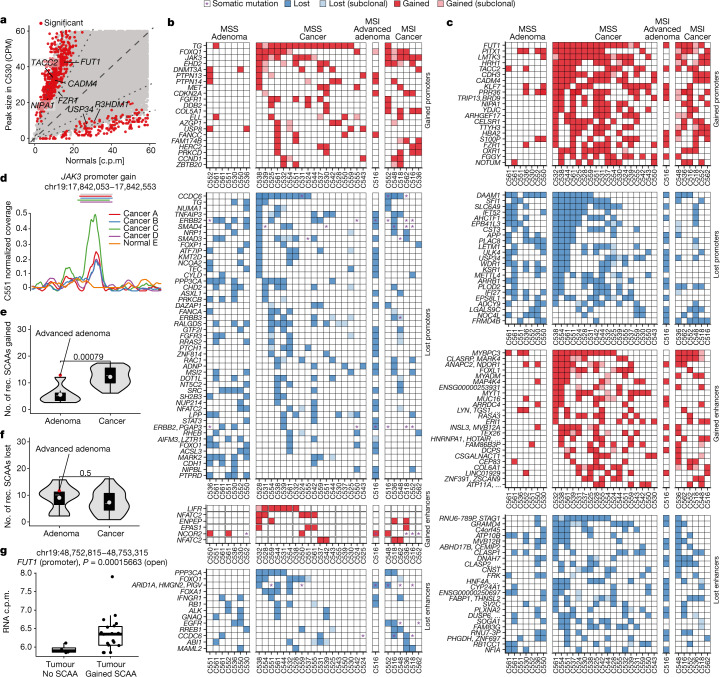
SCAAs in cancers and adenomas. **a**, Example of SCAAs detected in cancer C530 versus normal. Significantly altered peaks are shown in red. MS, microsatellite. **b**, SCAAs affecting known cancer driver genes occurring in ≥4 cases. Stars indicate DNA mutations found in the gene. **c**, Summary of the 25 most recurrent SCAAs in promoter and putative enhancers of genes not previously associated with cancer through DNA mutation. Subclonal changes are marked in shaded squares. **d**, Clonal somatic peak gained at the *JAK3* promoter in cancer C551. The plot shows the normalised peak coverage of glands from different regions (see colour legend). The coloured lines on top of the plot show called peaks and the grey line shows the interval of the reference peak. **e**,**f**, SCAA burden of adenomas versus carcinomas for gain (**e**) versus loss (**f**) of accessibility. The number of gains, but not losses, of accessibility differed significantly (two-sided *t*-test) between adenomas (*n* = 8) and cancers (*n* = 24) after subsampling the number of reads in carcinomas to those in adenomas. The lower and upper hinges of the boxes show the first and third quartiles. The whiskers extend to the largest and smallest value up to 1.5 times the interquartile range from the hinges, and values outside this range are shown as individual points. The grey horizontal lines within the boxes indicate the median, and the dots indicate the mean. The advanced adenoma of C516 is highlighted as a red dot. rec., recurrent. **g**, Example of a promoter for which we confirmed changes in gene expression. The gene expression between the groups of cancers with matched RNA-seq that showed evidence of accessibility gain (*n* = 18) and those that did not (*n* = 5) was compared using the DESeq2 contrast function.

**Extended Data Fig. 2:**
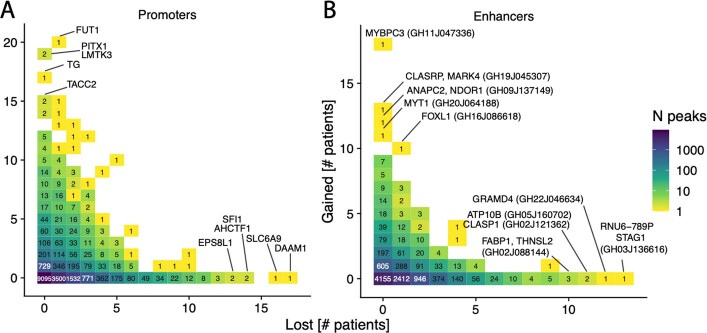
Recurrence of SCAA. A) Number of times each promoter and B) enhancer showed gained (y-axis) and reduced (x-axis) accessibility.

Recurrent genetic events in cancer driver genes clearly demonstrate the role of somatic alterations in cancer evolution, but is is unclear how common epigenetic changes of chromatin accessibility in CRC are. We examined the landscape of somatic chromatin accessibility alterations (SCAAs) in our cohort. We identified peaks in the ATAC-seq data for each region of a cancer using MACS2 (ref. ^34^) and compared each peak size in the tumour versus a pool of normal samples, while normalizing for the effect of CNAs (see figures at 10.6084/m9.figshare.19849789), to identify significant SCAAs (Fig. 3a and Methods). We found highly recurrent SCAAs in both promoters (Extended Data Fig. 2A) and putative enhancers (Extended Data Fig. 2B) of several genes of interest, including many previously associated with cancer. We note that these levels of recurrence are as high if not higher than for many genetic driver mutations (Fig. 2c).

Recurrent SCAAs were found in known cancer driver genes previously identified by genetic studies (Fig. 3b and list in Supplementary Table 5; shown are events occurring in ≥4 individuals). Many of these genes were devoid of genetic mutations in our cohort (marked with purple stars in Fig. 3b), confirming that SCAAs are an alternative modality for driver gene (in)activation. We also found recurrent SCAAs in genes that were not previously associated with tumorigenesis by means of genetic mutation (Fig. 3c, shown are the 25 most recurrent loci per group excluding those in Fig. 3b, example in Fig. 3d.

We then leveraged our spatial multi-region profiling strategy to assess intra-tumour SCAA heterogeneity. The signal from ATAC peaks is difficult to compare between samples because it is confounded by variability in purity and transcription start site enrichment. We used our matched WGS to identify clonal (truncal) DNA mutations present in all samples of the tumour and assessed the frequency of these variants in the reads from ATAC-seq to obtain accurate estimates of sample purity (Methods and Supplementary Table 2). Samples from each region were treated as pseudo-‘biological replicates’, and each of the signals for the different cancer regions was compared with that of the corresponding normal tissue while accounting for purity (Methods). A total of 24/30 cancers and 10/10 adenomas had sufficient samples with enough purity for the analysis. We focused on the 25 most recurrently altered loci per category (promoter or enhancer, gained or lost), as well as those associated with CRC driver genes found in ≥4 cases (Supplementary Table 6). We found that for most of these events (5,688/5,824, 97.7%), we had no evidence that they were subclonal, suggesting that most SCAAs are clonal epigenetic changes in the malignancy (Fig. 3b,c, see shading).

Among the recurrently altered and almost invariably clonal epigenetic changes, we found a *JAK3* promoter gain of accessibility in 11/24 cancers (Fig. 3d), as well as loss of chromatin accessibility in the CRC tumour suppressor gene *CCDC6*. This was the case for both the promoter (12/24 cancers) and an associated enhancer region (3/24 cancers); see, for example, case C524 in Supplementary Fig. 5A. Notably, mutations in *CCDC6* are infrequent in CRC (3/30 cases in our cohort, annotated as a purple star in Fig. 3b). Furthermore, *ARID1A* enhancer loss was observed in four cancers and one adenoma, with only two of those cases also bearing a mutation in this gene. Alterations in other putative CRC drivers were also found, such as *SMAD3* and *SMAD4* promoter loss, and *NCOR2* enhancer gain. *NFATC2* and *LIFR* cancer driver genes that were not reported in CRC were found to be epigenetically altered in our cohort, and in the absence of DNA mutations. Of interest, we found typically clonal promoter SCAAs in *FOXQ1* in 11/24 cases, a known oncogene reported to be involved in CRC tumorigenicity^35^, angiogenesis and macrophage recruitment during progression^36^. Although most recurrent SCAAs were clonal in the cancer, a proportion of SCAAs were found to be subclonal and confined to one or more regions. This was exemplified by a *FOXL1* enhancer gain (12/24 cases, 50%) in Supplementary Fig. 5B occurring only in regions C and D of cancer C524.

We note that ATAC peaks called in our dataset overlapped with peaks from the The Cancer Genome Atlas dataset that was composed of single CRC bulks^14^ and the ENCODE normal colon tissue dataset^37^. Moreover, the average peak sizes correlated strongly when reanalysed with our pipeline (Supplementary Fig. 6). Owing to unmatched normal controls however, in these orthogonal bulk-sample datasets it is complicated to distinguish chromatin changes that occurred in the cancer versus those present in the normal colon (for example, to determine the somatically changed status of the peak), and indeed most of the signal of chromatin accessibility comes from the tissue of origin of the sample^14^.

## Chromatin changes in adenomas and cancers

We then sought to determine the role of SCAAs in the adenoma–carcinoma transition, while not discarding the possibility that some of these changes may be a product of normal tissue ageing. We examined the stage of tumour development when SCAAs occurred. Out of the 665 recurrent SCAAs found in cancers (≥6 cases) with available concomitant adenomas, only 113 (17.0%) were also detected in the matched adenoma, suggesting that most SCAAs probably occurred at the onset of malignant transformation, hence after neoplastic growth initiation but before subclonal diversification (as they were also largely clonal). Such events are exemplified by the gain of accessibility of a *NXPH1* enhancer (4/24 patients, 17%) in C561, which was present in each region of the cancer but not in any of the concomitant two adenomas (Supplementary Fig. 5C and additional figures at for all events). Indeed, the lower SCAA burden of adenomas compared to that of cancers was not dependent on purity or read depth (Supplementary Fig. 7A,B). By explicitly normalizing for coverage (Supplementary Fig. 7C), we found a significantly lower burden of recurrent gain-of-accessibility SCAAs (>10 patients) between adenomas and carcinomas (Fig. 3e). No difference was found in the burden of loss of accessibility (Fig. 3f). We note that the only advanced adenoma in our cohort that was found co-locating with the cancer (C516; see Supplementary Fig. 1) indeed showed the SCAA gain burden of a carcinoma (Fig. 3e). It was previously noted that there were limited differences between adenomas and carcinomas in CRC at the level of point mutations in driver genes, and instead major differences at the level of chromosomal instability^3^. Here we additionally found differences in epigenetic rewiring between adenomas and cancers. Moreover, the higher burden of SCAA gains in cancers supports the idea that carcinogenesis involves an increased genome-wide chromatin accessibility.

To gain more insight on the origins of SCAAs, we investigated chromatin changes in the normal colon by comparing each normal crypt against the pool of normals from the other patients. We found very few SCAAs in individual normal crypts, supporting the idea that the SCAAs we observed in the tumours were indeed somatic alterations originated during tumourigenesis, rather than during the normal process of epigenetic ageing of colon crypts. A small subset of SCAAs was detected in multiple crypts of the same patient (Supplementary Figure 8A), but SCAAs in normal crypts were not recurrent (Supplementary Figure 8B) and did not overlap with SCAAs observed in tumours (Supplementary Figure 8C). Plausibly germline genetic variation could cause some of the chromatin accessibility alterations in normal tissue we observe.

## Impact of SCAAs on gene expression

We assessed the impact of SCAAs on gene expression using matched RNA-seq (for example, Fig. 3g). More than 10.8% of promoters (41/379) and 13.5% of enhancers (10/74) with recurrent SCAAs (≥6 patients) showed signs of altering the expression of associated genes (false discovery rate (FDR) < 0.01, Methods, Supplementary Table 7 and figures at 10.6084/m9.figshare.19857274). We note that chromatin accessibility measures the potential for transcription, indicating priming for future expression or a remnant ‘scar’ of past transcription. Therefore, more chromatin changes than those that correlate with expression in our analysis may actually be important for tumour evolution. Moreover, the power to detect expression changes was limited by the recurrence of a given SCAA in the cohort, incomplete matched RNA data and the lack of information about other factors influencing transcription such as methylation, post-translational modifications or *trans* regulation. To further probe the impact of somatic mutations on SCAAs, we analysed SNVs that we found were associated with changes in *cis* gene expression in our associated article^38^ and found that some of these SNVs co-occurred with a change in chromatin accessibility at the locus (figures at 10.6084/m9.figshare.19857274).

## Transcription factor signals indicate epigenetic reprogramming

**Fig. 4:**
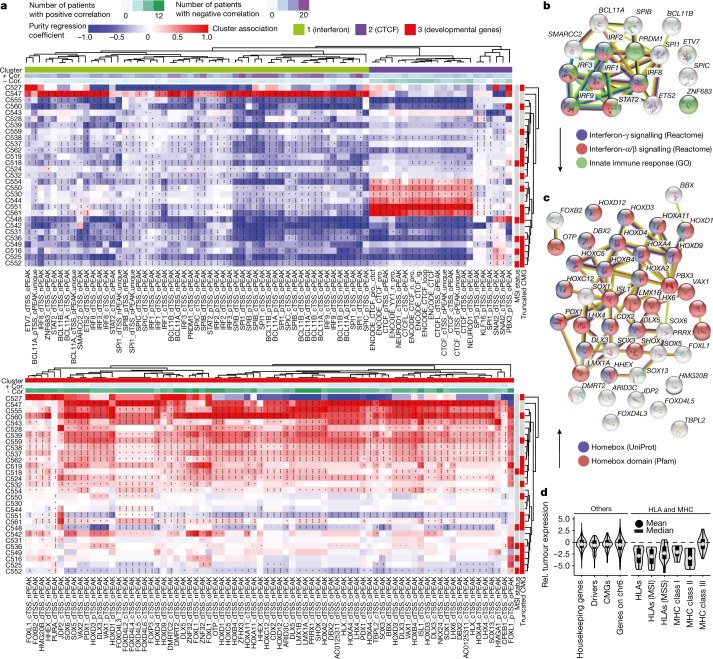
Accessibility of the TF-binding site is rewired in tumours. **a**, The differential signal across TF-binding sites between tumour and normal samples (see Extended Data Fig. 3) was regressed against transcription start site enrichment and purity to identify altered TF binding accessibility in tumours. Results are shown here for the three main clusters of differentially accessible TF loci (heatmap colour is the regression coefficient; star indicates significance). Main cluster identity is denoted by the top annotation columns. IRF, interferon-regulatory factor. **b**, STRINGdb analysis of the green TF cluster highlights downregulation of interferon signalling. GO, Gene Ontology. **c**, STRINGdb analysis of the red cluster indicates upregulation of the activity of developmental genes of the homeobox family. **d**, Relative (Rel.) tumour expression of HLA genes versus other gene groups. The lower and upper hinges of the boxes show the first and third quartiles. The whiskers extend to the largest and smallest value up to 1.5 times the interquartile range from the hinges, and values outside this range are shown as individual points. The grey horizontal lines within the boxes indicate the median, and the dots indicate the mean. Housekeeping genes from Ref. ^61^. CMGs, chromatin modifier genes. MHC, major histocompatibility complex.

**Extended Data Fig. 3:**
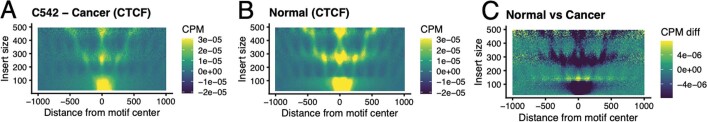
Transcription factor binding site accessibility is rewired in tumours. (**A**) TF binding site accessibility (in this example CTCF) is computed by summing the signal of ATAC-seq reads centred at the binding site, plotted against read length. (**B**) The same is done for the normal controls. (**C**) Signal from the normal is subtracted from the signal from the cancer to assess differential accessibility. TF accessibility for CTCF is reduced in this example as demonstrated by fewer ATAC cuts at the binding site in the cancer.

We extended our analysis beyond focal changes in chromatin accessibility in promoters and enhancers, investigating whether chromatin architecture could have a genome-wide influence on transcriptional control. To examine this, we analysed the genome-wide accessibility of transcription factor (TF) binding sites for 870 TFs^37^ using publicly available data for TF motifs and for chromatin immunoprecipitation followed by sequencing (ChIP–seq; Methods). We piled up the ATAC reads for all binding sites of a given TF across the genome and plotted read count versus the distance from the centre of the TF motif and the length of each read, producing a characteristic signature of TF accessibility for a given sample, which also encodes the footprint of the TF complex itself, in the cancer (Extended Data Fig. 3A and additional figures at 10.6084/m9.figshare.19857391) and normal (Extended Data Fig. 3B) regions. The normalized difference of the TF signal between tumour and normal glands indicated somatic changes in accessibility (Extended Data Fig. 3C). These analyses suggested pervasive genome-wide rewiring of TF chromatin accessibility in CRCs (Fig. 4a, see Methods for details). As many TFs bind to similar loci, we considered only largely non-overlapping TF annotations to ensure that a single locus could not drive the signal of several TFs (figures at 10.6084/m9.figshare.19857391).

Unsupervised clustering of somatic TF binding signatures produced three main clusters. The first main cluster (green cluster, Fig. 4a) seemed to be associated with downregulation of interferon signalling through loss of chromatin accessibility in loci putatively bound by TFs from the interferon-regulatory factor family, suggesting suppression of immune signalling. Reactome and Gene Ontology analysis (Fig. 4b) indicated that the signal was significantly enriched for downregulation of interferon-γ (FDR = 0.003) and interferon-α/β (FDR = 0.00075). This signal was stronger in MSI cancers, which are heavily infiltrated by immune cells (*P* = 0.012, Fisher’s exact test).

The second main cluster (blue cluster, Fig. 4a) contained two distinct subgroups of patients with differential chromatin accessibility for CTCF. The CCCTC-binding factor (CTCF) is a key player in chromatin insulation, determining looping and formation of the topological associating domain. Most cases were characterized by loss of accessibility of the CTCF-binding site, particularly in MSI cancers. A smaller group showed increased CTCF accessibility. CTCF chromatin accessibility alterations were previously noted in single-bulk cancer samples^39^. *CTCF* somatic mutations can occur in CRC^40^, and indeed a mouse model of chronic *CTCF* hemizygosity led to higher cancer incidence and dysregulation of oncogenic pathways^41^.

The third main cluster (red cluster, Fig. 4a) showed increased chromatin accessibility for TFs involved in development, such as the HOX, FOX and SOX families (UniProt: ‘homeobox’, FDR = 0.00069). The chromatin accessibility of this cluster of TFs was higher in cancer in most cases, suggesting possible reactivation of developmental genes in CRC tumorigenesis (Fig. 4c). The expression of the TFs involved in this cluster is reported in Supplementary Fig. 9.

Notably, matched RNA-seq data showed that gene expression of human leukocyte antigen (HLA) genes was significantly reduced in both MSS and MSI cancers with respect to normal samples (Fig. 4d) consistent with the downregulation of interferon signalling as highlighted by the signal in the green cluster.

We also noted a small cluster characterized by increased accessibility at the TF-binding sites of SNAI1 and SNAI2, two TFs involved in epithelial-to-mesenchymal transition^42^. This cluster was significantly enriched in cases showing truncating mutations in chromatin modifier genes (*P* = 0.047, Fisher’s exact test), consistent with previously reported regulation of epithelial-to-mesenchymal transition by chromatin modulators^43^. We cannot exclude that there could be further subgroups of patients with distinct TF accessibility patterns beyond the CTCF subgroup (blue cluster); further studies with more patients are needed.

## Demethylation of developmental TF-binding sites

We further attempted to corroborate the increased accessibility to TF involved in development. Changes in chromatin accessibility can be accompanied by changes in DNA methylation, with heterochromatin regions often being methylated and vice versa for open chromatin regions. This is particularly the case for regions that are permanently silenced after development^44^. We tested whether SCAAs identified at TF-binding sites (Fig. 4a) were reflected in the methylation of the same loci. We performed methylation profiling on a subset of 8 samples using Illumina EPIC 850k methylation arrays (one sample from C516, two samples from C518, two samples from C560 and three samples from C561; see Methods for details). First, we report that C518 is probably a CpG island methylator phenotype case according to established markers^45^ (Supplementary Fig. 10). Comparing the methylation of TF binding annotations in cluster 3 (Fig. 4c), methylation in these regions was found to be significantly lower than that in normal tissue, supporting the finding that these sites were accessible (Supplementary Fig. 11a). This was particularly clear for TF-binding sites of DLX5, HOXA4, HOXB4, ISL1, SOX5 and SOX6 (Supplementary Fig. 11b), suggesting stable reactivation of regulatory regions involved in developmental genes. We note that this was not due to a general pattern of global hypomethylation, as methylation in genes that are usually normally highly methylated in normal were also high in cancer (Supplementary Fig. 12).

## Chromatin changes are stable and heritable

Epigenetic alterations, and in particular chromatin modifications, are responsible for cell identity in all tissues, but it remains unclear whether epigenetic changes in cancer are stable during tumour evolution. Seminal studies have begun unravelling epigenetic heritability in blood cancers^46,47^, and suggest that stable SCAAs could provide a heritable substrate for Darwinian selection to operate. For most detected SCAAs, if the peak was differentially accessible in one region of the tumour, it was also differentially accessible in other distant regions. As we sampled opposing tumour sides (Fig. 1e,f), two sampled regions likely have early common ancestors, diverging by a large number of cell divisions. Hence, we argue that most SCAAs we detect are probably clonal or have high ‘clonality’ (that is, they are shared by large proportions of cancer cells). This can occur either through convergence of different lineages to the same SCAAs, or through evolution by common descent. Given the number of putatively clonal SCAAs, as well as the distance and the probable difference in microenvironment between the distinct regions of each cancer, we argue that the most parsimonious explanation is, as for species evolution, evolution by common descent, rather than convergence of many different lineages to the same overall epigenetic pattern.

To further test the heritability of epigenetic alterations, we specifically compared SCAAs within versus between tumour regions (Supplementary Fig. 13A). In most patients (23/29), analysis of variance controlling for transcription start site enrichment and total read count showed that samples from the same region were significantly less divergent in terms of SCAAs than samples from different regions (Supplementary Fig. 13B). Moreover, a direct correlation between genetic distance and epigenetic distance was found in 8/29 cases (the power of this analysis is limited by small sample numbers), after controlling for purity (see the example in Supplementary Fig. 13C). This was not the case for all patients, either because of lack of a correlation or not enough data (see the example in Supplementary Fig. 13D). Thus, chromatin profiles were heritable and followed, at least in part, genetic divergence (Supplementary Fig. 13B; see coefficients of the analysis of variance per region in Supplementary Fig. 14), thus providing further evidence that common descent, not convergence, is the reason for SCAAs common to several samples of the same tumour. Genome-wide TF SCAAs (Fig. 4) showed similar evidence of heritability (figures at 10.6084/m9.figshare.19857391), suggesting that such rewiring of the chromatin existed in a common ancestor of all the samples and was inherited during tumour growth. There were however some interesting exceptions in which different regions showed distinct SCAA profiles. For example, whereas C548 showed homogeneous loss of accessibility to CTCF-binding sites at loop loci, in C543 both promoter- and loop-binding sites of CTCF were altered and in a heterogeneous manner, with regions exhibiting differential organization of the chromatin (figures at 10.6084/m9.figshare.19857391).

## Mutational signatures affecting the epigenome

There is a growing appreciation of the multidimensional nature of mutation signatures beyond the 96-channel representation and across different regions of the genome, especially in relation to replication time and three-dimensional genome organization^48^. However, the relation between mutational signatures and epigenetic features remains poorly studied owing to lack of matched data. Here we examined the feedback between epigenome and transcription status and mutational processes^49,50^ through tumour evolution. We performed de novo signature discovery using a methodology robust to overfitting^51^, detecting six mutational signatures across our cohort (Supplementary Figs. 15A and 16): SparseSignature1, corresponding to COSMIC signature 1 of C>T deamination at methylated CpG sites; SparseSignature2, corresponding to COSMIC signatures 2 and 13 caused by APOBEC enzymes; SparseSignature3, corresponding to COSMIC clock-like signature 5; SparseSignature4, corresponding to COSMIC signatures 17a and 17b of unknown aetiology; SparseSignature5, corresponding to COSMIC signatures 9 and 41, also of unknown aetiology; SparseSignature6, corresponding to COSMIC signature 44 caused by mismatch repair deficiency.

**Fig. 5:**
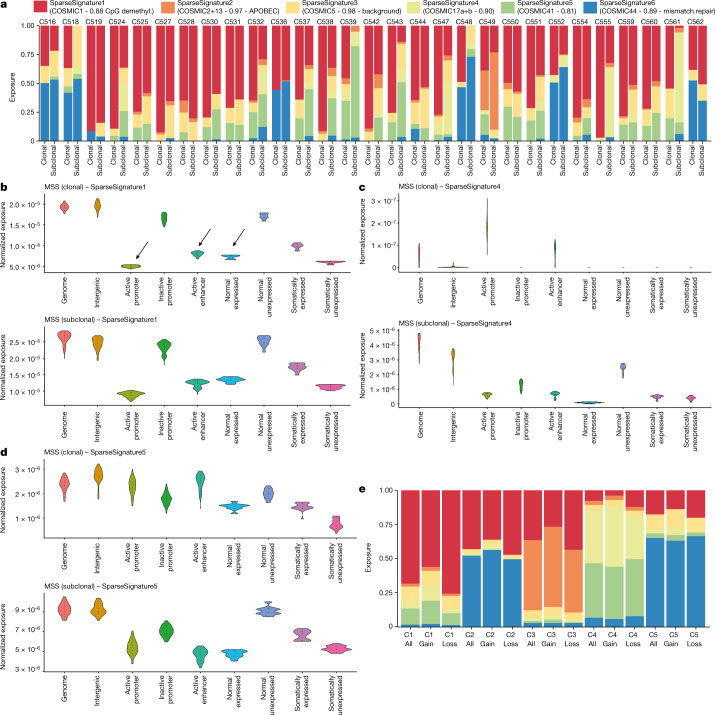
DNA mutational signatures and the epigenome. **a**, Clonal and subclonal mutational signature composition for each case. CpG demethyl., CpG demethylation. **b**, The epigenome influences accumulation of deamination signature 1 in distinct regions, both for clonal and subclonal mutations. **c**, Signature SparseSignature4, mostly present subclonally, is also influenced by the epigenome status. **d**, Signature SparseSignature5, particularly at the subclonal level, is again depleted in active regions as SparseSignature1. **e**, The proportion of each signature for every cluster responsible for generating loss or gain of CTCF binding affinity in our cohort.

Genome-wide signature activity divided the cohort into five distinct clusters of patients (Supplementary Fig. 15B,C). The two main clusters consisted of MSS (cluster 1) and MSI cases (cluster 2). Cluster 3 contained only case C549, which was strongly enriched with the APOBEC signature. Cluster 4 with cases C561 and C539 had high activity of SparseSignature4 and SparseSignature5 of unknown aetiology. Cluster 5 with cases C518 and C548 had higher SparseSignature3 (clock-like signature). We assessed changes in mutational process activity over time by comparing inferred activity between clonal and subclonal mutations (Fig. 5a). SparseSignature1 (deamination) was dominant in MSS cases throughout tumour evolution, and in MSI cancers SparseSignature6 (mismatch repair) was also dominant throughout. SparseSignature2 (APOBEC), SparseSignature4 and SparseSignature5 (unknown) were enriched at the subclonal level in cases in which they were active, demonstrating activity late in tumour evolution.

Mutations in chromatin modifier genes, or alterations in TF-binding sites, can determine the characteristics of the epigenome. Conversely, chromatin architecture determines how the cancer genome accumulates mutations owing to its effect on different mutational processes and activity of DNA repair genes^52,53^. To examine the impact of the epigenome on the accumulation of mutations further, we compared mutational signature burdens between epigenetic regulatory regions identified with the ATAC-seq data (active and inactive promoter, active and inactive enhancer, intergenic, and coding), as well as typically expressed and not expressed genes identified with the RNA-seq data.

SparseSignature1 (cytosine deamination) was 2–4-fold higher in closed chromatin regions of the genome (inactive promoters and enhancers) for both clonal and subclonal mutations, consistent with the need for methyl cytosine (enriched in inactivated regulatory regions) to be present for it to become deaminated and produce the associated mutational signature (Fig. 5b). Analogous differences were observed in the coding regions of the genome between genes expressed versus not expressed genes in the normal: specifically, genes that were ‘switched on’ in tumour after being off in normal carried an intermediate load of C>T deamination mutations that were probably accumulated in the normal tissue before carcinogenesis when the locus had inaccessible chromatin, before the mutation rate was reduced when the chromatin opened and gene expression was induced (Fig. 5b). Similar dynamics were observed for SparseSignature4 (Fig. 5c) and SparseSignature5 (Fig. 5d; ref. ^54^). The activity of the mismatch repair signature in MSI cases was more uniformly distributed across the genome (Supplementary Fig. 17).

We reasoned that different mutational processes may also differentially alter the affinity of the TF-binding site, as an example mechanism of how mutational processes can directly influence the cancer epigenome. It has previously been documented that point mutations can disrupt CTCF-binding sites^40^. We selected CTCF sites with somatic mutations that were predicted by deltaSVM^55^ to cause significant loss or gain of binding and assessed the relative contribution of each mutational signature to these mutations in the CTCF-binding site across the five mutational signature clusters. In MSS cancers (cluster 1), mutations predicted to cause loss of binding had a signature that was consistent with the background mutational signature acting on the genome (cosine similarity = 0.977; Supplementary Fig. 18A), and the same was true for gains (cosine similarity = 0.919; Supplementary Fig. 18B). In MSI cancers (cluster 2), SparseSignature6 (mismatch repair; Supplementary Fig. 18C) was consistent with causing gain of CTCF binding affinity (cosine similarity = 0.925). In C549, the only case with high levels of SparseSignature4 (COSMIC signature 17; Supplementary Fig. 18D), this signature was also a source of mutations causing gain of affinity (cosine similarity = 0.977). These results suggest that CpG deamination causes the largest proportion of mutations altering CTCF binding in MSS cancers, with a higher tendency of generating loss of binding (Fig. 5e). In MSI cases, the mismatch repair signature is also a dominant factor in causing altered binding of CTCF, with a preference for generating increased affinity (Fig. 5e). When considering the abundance of any given mutational signature in the genome, we found that 4% and 8% of SparseSignature1 mutations cause, respectively, gain and loss of CTCF binding, whereas 5% and 8% of SparseSignature6 mutations cause, respectively, gain and loss of CTCF binding (see all in Supplementary Fig. 19).

## Discussion

The contribution of epigenetic events to cancer evolution is recognized as highly significant^7,56^, but has remained understudied^5^. Recently, a pan-cancer analysis revealed the chromatin accessibility profile of several cancer types, but the lack of an appropriate matched normal control precluded proper identification of cancer-specific events, with tissue-specific and ‘cell of origin’ chromatin profiles remaining the dominant signal in the data^14^. Studies with normal tissue references have identified complex patterns of SCAAs in CRCs^12,13^, but have not been able to assess the evolutionary dynamics that led to these chromatin changes. Here we show that genetic and epigenetic modification of cancer-associated genes occurs independently but recurrently in CRCs, and that epigenome alterations probably control important tumour cell phenotypes, including escape from immune surveillance. Further, we find that chromatin alterations are stable and heritable, providing a substrate for Darwinian selection to act, and interrelatedly, chromatin alterations influence the accumulation of somatic genetic alterations that can also drive evolution^57,58^. At present, genomics detects driver alterations or mutational processes that inform on drug sensitivity but is blind to potentially clinically actionable biology governed by the epigenome. The observation that epigenetic changes occur in regulatory regions of known cancer driver genes in the absence of somatic mutations argues for the importance of epigenomics for genomic medicine. Certainly, the interaction between somatic mutations and SCAAs remains challenging to unravel. Although several studies have investigated the effects of somatic mutations in chromatin modifier genes (for instance, linking mutations with increased transcriptional heterogeneity^59^), identifying the direct (*cis*) functional effects on the chromatin caused by DNA variants remains difficult. Our multi-omic dataset provides some clear examples of a genome–epigenome relationship: we observed somatic mutations associated with both changed *cis* gene expression and changed chromatin accessibility. Follow-up work is required to explore the functional impact of epigenetic alterations in cancer driver genes and other loci.

We also observed that the epigenomes of adenomas and carcinomas are distinct. The lower prevalence of SCAAs in adenomas and, at the same time, the clonality of most SCAAs in carcinomas suggest that many cancer SCAAs may occur at the onset of malignant transformation. This is important because, besides broad CNAs, mostly non-focal chromosomal arm gains or losses of unknown significance, there is little difference in driver alterations between benign adenomas and malignant carcinomas^3^. Moreover, there is no validated prognostic genetic alteration that predicts recurrence in CRC. Others have shown that chromatin topology changes over time in ageing colon tissue, including in transformed tissues, and that a link between altered chromatin patterns and patient outcomes exists^60^. This is consistent with our finding of a decisive role for SCAAs in cancer biology. We acknowledge that our multi-omic analysis was based on the analysis of tumour glands, and it possible that the biology could differ in the rare CRCs that completely lack glands.

One of the most intriguing results was the evidence of reactivation of developmental genes during tumorigenesis. Those genes are usually silenced in somatic tissue, and the reactivation of the genes in these families and their involvement in tumorigenesis has been postulated before in the context of glioblastoma tumorigenesis^54^ as an enabler of growth and adaption. We identified a group of TFs with decreased accessibility that were related to interferon signalling. On the other hand, we also found a group of TFs that had increased accessibility and was enriched with homeobox genes (for example, *SOX5* and *SOX6*) that are directly involved in early development. We speculate that we may detect biological processes that aim at reprogramming cell fate through reactivation of developmental genes. Further functional work is warranted.

Overall, our spatially resolved multi-omic analysis of primary colorectal cancers shows non-genetic determinants of cancer cell biology and clonal evolution.

## Methods

### Sample collection

Primary tumour tissue and matched blood samples were prospectively collected from patients undergoing curatively intentioned surgery at University College London Hospital (UCLH). All patients gave informed consent for collection of their materials to the UCLH Cancer Biobank (Research Ethics Committee approval 15/YH/0311). Four regions of each primary cancer were sampled by punch biopsy or scalpel dissection, at notionally 12, 3, 6 and 9 o’clock positions around the tumour periphery. Each region was further cut into four pieces and slow-frozen to −80 °C, using a Mr Frosty Freezing Container (Thermo Fisher), in 1 ml of a buffered medium (MEM supplemented with 5% FBS and 0.5% 5 mM HEPES buffer, diluted with 10% dimethylsulfoxide) in a 1.8-ml Nunc Cryotube (Sigma-Aldrich), and immersed in isopropanol to preserve chromatin structure. All investigators were blinded to patient data related to outcome, gender and other clinicopathological information.

### Gland isolation

A clean glass slide was placed into a 10-cm Petri dish and 500 μl PBS supplemented with RNAse and protease inhibitors was pipetted on top of the slide. The Petri dish was then transferred to the stage of a dissecting microscope. Tissue pieces were manually dissociated under the microscope using two 16G needles, with individual glands being pulled away from the tissue mass. For every specimen, a further epithelial minibulk sample that comprised a total of approximately 10–20 crypts or glands was collected. Each gland was transferred to a 1.5-ml Eppendorf tube containing a total volume of 50 µl cell lysis buffer (10 mM Tris-HCl pH 7.4, 10 mM NaCl, 3 mM MgCl_2_, 0.1% IGEPAL CA-630 supplemented with protease inhibitors (1 tablet in 50 ml dH_2_O as directed by the manufacturer (cOmplete Protease Inhibitor Cocktail, Sigma-Aldrich) and RNASE inhibitor 1 U µl^−1^ (Protector RNase Inhibitor, Sigma-Aldrich) and incubated on ice for 10–45 min. Bulk samples were collected in a final volume of 100 µl cell lysis buffer. We found that longer or warmer incubations decreased the RNA quality and yields and negatively affected chromatin structure. In selecting the 30 cases included in our study, we rejected only a single further case owing to being unable to isolate any glands, confirming that retention of glandular structures is pervasive in CRC.

### Chromatin, DNA and RNA separation

Each tube containing an individual gland or bulk was lightly vortexed, transferred to a pre-chilled centrifuge and spun at 500*g* for 10 min holding the temperature at 4 °C. This produced a pellet of cell nuclei at the bottom of the tube, with the cytosolic fraction present within the supernatant. For RNA extraction, 45 µl (glands) or 90μl (bulks) of the supernatant was transferred into a new tube containing 300 µl of TRIzol (taking care not to disturb the pellet). TRIzol lysates were stored at −20 °C if not processed immediately or at −80 °C for long-term storage. For extraction of nuclear material, the pellet of nuclei was resuspended in residual cell lysis buffer. A 2.5 µl volume of suspension was transferred into another tube for subsequent DNA extraction, which was frozen if required. The remaining suspension was immediately used for preparation of ATAC-seq libraries, as we found subsequent handling or storage compromised library quality.

### Preparation of ATAC-seq libraries

Tubes containing the suspension of nuclei (2.5 µl for glands and 7.5 µl for minibulks) were kept on wet ice. A 2.5 µl volume of 2× TD buffer and 0.25 µl of Tn5 transposes (Illumina) was added to each gland tube and 25 µl 2x TD buffer, 2.5 µl Tn5 transposes and 15 µl of DNAseq/RNase free water was added to each minibulk tube before incubation at 37 °C for 30 min. Purification was performed using AMpure XP SPRI beads (Beckman Coulter); 10 µl (2× sample volume) of room-temperature beads was added to each tube and mixed by pipetting 10 times, before incubation at room temperature for 1 min. The tube was placed on a magnetic plate, and beads were allowed to settle for 3 min. Once clear the supernatant was discarded. With the tube still on the magnetic plate, 200 μl of 80% ethanol was added and incubated at room temperature for 30 s, the ethanol supernatant was discarded. The tube was removed from the magnetic plate and 10 µl of 10 mM Tris buffer was added to each tube and mixed. The tubes were placed on a magnetic plate, and the beads were allowed to settle for 3 min. Once clear, 10 µl of supernatant containing purified transposed DNA fragments was transferred to a fresh tube for immediate library preparation or stored at −20 °C for later use.

For library preparation, the transposed sample was supplemented with 1 µl of 10 µM Nextera i7 PCR primer, 1 µl of 10 µM Nextera i5 PCR primer (Illumina) and 12.5 µl of NEBNext Q5 High-Fidelity 2× PCR Master Mix (New England Biolabs). PCR amplification was performed, with initial elongation at 72 °C for 5 min, then initial denaturation at 98 °C for 30 s, and then 14 cycles (for glands) or 10 cycles (for bulks) of the following: 10 s of denaturation at 98 °C, annealing step at 63 °C for 30 s followed by 72 °C for 1 min.

Following amplification, samples were purified with 2× SPRI beads and eluted in 20–30 µl of 10 mM Tris buffer, pH 8. Samples were screened using the Agilent Tapestation 4200 and HSD1000 screentapes. Only those that showed a fragment size distribution with peaks at multiples of about 147 base pairs (bp), indicating intact nucleosomal structure within the nuclei, were sent for sequencing.

### Preparation of WGS libraries

DNA fractions were extracted using the Zymo QuickDNA Microprep plus kit according to the manufacturer’s instructions. Only samples with a total DNA yield higher than 10 ng were taken forwards for WGS library preparation. Libraries were prepared using the NEBNext Ultra II FS kit according to the manufacturer’s instructions. A short enzymatic fragmentation step of 5 min was performed, and five PCR cycles were used for library enrichment. After purification, libraries were quantified by Qubit and run on the Agilent Tapestation using HSD1000 screentapes. Samples with sufficient library DNA yield and characteristic fragment size distribution (about 200–500 bp) were further subjected to either low-pass (about 1× coverage) or deep (about 35× coverage) WGS.

### RNA library preparation

The cytoplasmic fractions of each sample in the form of TRIzol lysates were used for RNA extraction using the Directzol kit (Zymo R2052). Modifications to the manufacturer’s protocol were introduced to increase the total RNA yields. First, we passed the initial TRIzol and ethanol mix twice through the spin column. Second, we eluted the RNA using two 25 µl volumes of water instead of just one 50 µl elution. The optional DNase step was used.

Agilent Tapestation quality control showed low RNA integrity number scores (<3) for most samples and so was not used to exclude samples for library preparation. Libraries were prepared using the Illumina TruSeq RNA Exome kit (compatible with low-quality input material) according to the manufacturer’s instructions.

### Methylation arrays

DNA methylation array analyses were carried out on selected bulk samples with sufficient DNA yield. Genomic DNA was bisulfite-converted using the Zymo EZ DNA Methylation kit. A 50-µl reaction containing 2.5–100 ng of DNA was incubated in the dark using a modified conversion protocol: 95 °C for 30 s and then 50 °C for 60 min, for 16 cycles and then holding at 4 °C. The full 8 µl eluate of converted DNA was repaired using the Infinium HD FFPE Restore Kit (Illumina). All 8 µl of the bisulfite-converted DNA for each sample was analysed on the lllumina Human MethylationEPIC BeadChip (Illumina). Processing was carried out by the University College London Genomics Core Facility according to a standard protocol.

### Sequencing

Sequence libraries were multiplexed and sequenced on an Illumina NovaSeq, typically using S2 flow cells. Read length and depth were varied as required by library composition. Sequencing was performed by the Institute of Cancer Research Tumour Profiling Unit.

### Alignment for WGS

Contaminating adapter sequences were removed using Skewer v0.2.2 (ref. ^62^). Adapter sequences were 5′-AGATCGGAAGAGC-3′ and 5′-ACGCTCTTCCGATCT-3′, with a maximum error rate of 0.1, a minimum mean quality value of 10 and a minimum read length of 35 after trimming using the options -l 35 -r 0.1 -Q 10 -n. The trimmed and filtered reads from each sequencing run and library were separately aligned to the GRCh38 reference assembly of the human genome^63^ using the BWA-MEM algorithm v0.7.17 (ref. ^64^). Following the GATK best practices and the associated set of tools v4.1.4.1 (refs. ^65–67^), reads were sorted by coordinates (GATK SortSam), independent sequencing runs or libraries generated from the same tissue sample were merged and duplicate reads were marked using GATK’s MarkDuplicates. The structure of the final bam files was verified using GATK’s ValidateSamFile.

### Alignment for ATAC-seq

Adapter sequences were removed with Skewer v0.2.2 (ref. ^62^) using the following full-length adapter sequences with the option ‘-m any’: 5′-CTGTCTCTTATACACATCTCCGAGCCCACGAGACNNNNNNNNATCTCGTATGCCGTCTTCTGCTTG-3′ 5′-CTGTCTCTTATACACATCTGACGCTGCCGACGANNNNGTGTAGATCTCGGTGGTCGCCGTATCATT-3′.

The reads of each sequencing run and library were aligned to the GRCh38 reference genome using Bowtie2 v2.3.4.3 (ref. ^68^) with the options ‘--very-sensitive -X 2000’ set. After sorting the reads with SAMtools v1.9 (ref. ^69^), those mapping to non-canonical chromosomes and mitochondria (chrM) were removed (GATK PrintReads followed by RevertSam and SortSam). After merging independent libraries for each sample, we removed duplicate reads using GATK’s MarkDuplicates and removed all reads mapping to several locations (multi-mappers). The final bam files were validated with GATK’s ValidateSamFile.

### Detection of germline variants

HaplotypeCaller v4.1.4.1 with the GATK package^70^ was used to identify germline variants from the reference normal samples in each patient (buffy coats or adjacent normal tissue) using known germline variant annotations from build 146 of the dbSNP database^71^ separately for each chromosome. Resulting VCF files were then merged with GATK MergeVcfs. Variant recalibration was performed with GATK’s VariantRecalibrator with options set according to GATK best practices^71–74^ and applied to VCF files using GATK ApplyVQSR with the options ‘-mode SNP -ts-filter-level 99.0’ and ‘-mode INDEL -ts-filter-level 99.0’, respectively. All germline variant calls marked as PASS were retained.

### Verification of sample–patient matches

For all samples, we excluded the possibility of sample mismatch by comparing germline variants identified in normal tissue to neoplasia samples of a given patient. The reads of each read group were extracted with SAMtools view using the options ‘-bh {input_bam} -r {read_group_id}’, and GATK’s CheckFingerprint tool was applied to extract statistics on sample–patient matches^75^. For virtually all high-purity samples without extensive loss of heterozygosity, we were able to confirm that the samples were obtained from the expected patient. A few samples with high amount of LOH and high purity fingerprinting did not confirm the sample-patient match; for these we instead inspected copy-number profiles (see below) to confirm that these matched the remaining samples of the corresponding patient.

### Copy-number analysis

#### Deep WGS

Coverage values for genomic loci relative to matched normal tissue samples (buffy coats or adjacent normal tissues) were extracted with methods provided in the Sequenza v2.1.2 package for R (ref. ^76^) and binned in non-overlapping windows of 10^6^ bp. B-allele frequencies of germline mutations determined with the GATK HaplotypeCaller (see above) for each patient were added to these binned files. Joint segmentation on B-allele frequencies and depth ratios across all samples from a given tumour were used to determine a set of breakpoints to use for the subsequent analysis. Specifically, GC content bias correction was applied using the gc.norm method from Sequenza v2.1.2, and positions with non-unique mappability (that is, <1), as determined by the approach of QDNAseq v3.8 (ref. ^77^), in windows of 50 bp were removed. Piecewise constant curves were fitted for each chromosome arm using the multipcf function (gamma = 80) from the copynumber v1.22.0 package for R (ref. ^78^). The per-patient set of breakpoints, binned depth ratio and B-allele frequency data were then inputted into the Sequenza algorithm (v2.1.2) to determine allele-specific copy numbers, ploidy *Ψ* and purity *ρ* estimates^76^. The initial parameter space searched was restricted to {*ρ* | 0.1 ≤ *ρ* ≤ 1} and {*Ψ* | 1 ≤ *Ψ* ≤ 7}. On manual review of the results, we identified several samples with unreasonable fits (cases in which calls suggested extremely variable ploidy values across samples). For these samples, we manually identified alternative solutions consistent with the other samples and somatic variant calls.

#### Low-pass WGS

Low-pass WGS bam files were processed using QDNAseq^77^ to count reads in 500-kilobase (kb) bins across the autosomes of hg38 and convert read counts into log_2_-ratios. Data normalization was performed in accordance with the QDNAseq workflow, except for outlier smoothing (smoothOutlierBins function), which was seen to artificially depress the signal from highly amplified bins. Bins for hg38 were also generated according to QDNAseq instructions. log_2_[ratio] values in each bin were normalized by subtracting the median log_2_[ratio] from all log_2_[ratio] values per sample. Samples from a patient were segmented jointly using the multipcf function in the R package copynumber (gamma = 10)^78^, and the mean segment log_2_[ratio] was calculated across the bins.

Absolute copy-number status was calculated using the approach taken by ASCAT^79^. Using the ASCAT equation to describe log_2_[*R* ratio] values, we took an integer ploidy value *Ψ*_*t*_ in the tumour *t* as determined by paired deep WGS in each case and searched a range of purities from 0.1 to 1 (and assumed gamma was 1 as is the case in sequencing data). For each purity (*ρ*) value, we calculated the continuous copy-number status of each bin and calculated the sum of squared differences of these values to the nearest positive integer of the modulus. Purity estimates were given by local minima (goodness of fit to integer copy-number values, measured as the sum of squared differences) across the purity range considered. The absolute copy-number state for each bin was taken as the closest integer value calculated using this purity. If no local minimum was found the purity was assumed to be 1. If the best solution produced negative copy-number states at some loci, these were set to have a copy number of zero to avoid impossible copy-number states. In two patients, per sample ploidies were determined by manual adjustment owing to integer ploidy values producing poor fits.

### SNV detection

Somatic mutations were first called for each tumour sample separately against matched blood derived buffycoats or adjacent normal tissue samples with Mutect2 (v4.1.4.1) using the options ‘--af-of-alleles-not-in resource 0.0000025 --germline-resource af-onlygnomad.hg38.vcf.gz’ (refs. ^70,80^). Variants detected in any tumour sample (marked PASS, coverage AD 10 in both normal and tumour, at least 3 variant reads in the tumour, 0 variant reads in the normal, reference genotype in normal and non-reference genotype in cancer) were merged into a single list of candidate mutations. The multi-sample caller Platypus v0.8.1.1 (ref. ^81^) was then used to recall variants at each candidate mutation position in all samples of the patient. In practice, this meant that the pipeline leveraged information across samples to improve the sensitivity of variant calling. The Platypus output of joint variant calls was then filtered to keep only high-quality variants with the flags PASS, alleleBias, QD or Q20, in canonical chromosomes (that is, not in decoy), a minimum number of reads NR > 5 in all samples, a genotyping quality GQ > 10 in all samples, a reference genotype (that is, 0/0) in the normal reference and a non-reference genotype (that is, 0/1 or 1/1) in at least one tumour sample.

To alleviate concerns of false-negative calls of mutations in important driver alterations, we generated a second set of variant calls for the identification of known driver mutations and d*n*/d*S* analysis (see details below) to which we did not apply the second step of filtering.

### SNV annotation

Somatic variants were annotated and candidate driver genes of CRCs reported by ref. ^3^ and IntOGen^82^ as well as pan-cancer driver genes reported by refs. ^33,83^ were filtered with the Variant Effect Predictor v93.2 (ref. ^84^).

### MSI status detection

The identification of MSI CRCs was performed with MSIsensor v0.2 (ref. ^85^). We first determined the position of microsatellite sites by applying the MSIsensor scan method to the GRCh38 reference assembly and subsetting the identified microsatellites to those located on the first chromosome. In a second step, we identified the fraction of mutated microsatellites in each sample using the MSIsensor msi method with default options. Generally, in known MSI cases (for example, those identified by mutation burden and mutational signature), more than 30% of microsatellites were mutated, and we used this as a critical value to classify cases as MSS and MSI. One exception was C562, in which the low purity of the samples led to a low MSIsensor score. However, this case was clinically classified as MSI by pathological reports, and it had a relatively high indel burden leading to the conclusion that it was MSI.

### Extraction of reads supporting variants

Using the VCF files from both somatic and germline variant calling, we extracted the number of reads supporting the reference and alternative alleles as well as the total number of reads covering the sites from WGS, low-pass WGS and ATAC-seq samples using Python and the Pysam library^69^, Pysam v0.15.2, SAMtools v1.9.

### d*n*/d*S* analysis

The dndscv package for R (ref. ^33^) was used for d*n*/d*S* analysis. Per-patient variant calls were obtained from the VCF files^86^ and lifted over to the hg19 reference genome using the rtracklayer package for R (ref. ^87^). Variants were divided into clonal mutations (that is, present in all samples) and subclonal mutations (that is, present in a subset of samples) present in the cancer and a set of mutations present in any of the adenoma samples. MSI and MSS cases were treated separately. dndscv was applied separately to each of the four sets (MSI or MSS and clonal or subclonal) (using default parameters apart from deactivated removal of cases because of a highe number of variants). Further, d*n*/d*S* values for a set of 167 chromatin modifier genes were extracted.

### ATAC-seq

#### Extraction of cut sites in ATAC peak-calling analysis

For the detection of cut sites (hereafter ‘peaks’ where read density was high), bed files of ATAC-seq cut sites were produced. Aligned reads were sorted by read name using SAMtools sort -n{bam}, and all proper reads pairs (that is, reads mapped to the same chromosome and with correct read orientation) were isolated using SAMtools view -bf 0x2 and finally converted to the bed format using bedtools bamtobed -bedpe -mate1 -i{bam}. As in ref. ^88^, the start site of reads was shifted to obtain the cut sites: specifically, forward reads were shifted by −4 bases and reverse reads were shifted by +5 bases. ATAC-seq reads spanning nucleosomes have an insertion size periodicity of multiples of 200 bp, and reads in regions of open chromatin have insertion sizes smaller than 100 bp (ref. ^88^). For this reason, as in previous studies, ATAC-seq reads were divided into a set of nucleosome-free reads (insertion size ≤ 100) and a set of nucleosome-associated reads (180 ≤ insertion size ≤ 620).

#### Peak detection in ATAC peak-calling analysis

Peaks were called separately for each tumour region using MACS2 v2.21 (ref. ^89^) using ‘macs2 callpeak -f BED -g hs --shift --75 --extsize 150 --nomodel --call-summits --keep-dup all -p 0.01’ with the concatenated and sorted bed read files of nucleosome-free cut sites of all samples as input. A set of normal peaks (across patients) was called using the concatenated normal sample bed files (that is, region E samples) as input. Per-adenoma peaks were called using all adenoma bulk samples as input.

#### Filtering and concatenation of peaks in ATAC peak-calling analysis

Per region peak calls were filtered for those having a *q*-value < 0.1%, enrichment > 4.0, and a maximum of the top 20,000 peaks. Iterative merging was then applied, using a method equivalent to that used in ref. ^11^ on per-region peak calls of individual patients (per-tumour peaks set) as well as across all cancer samples and pan-patient normal peak calls (pan-patient peak set). The iterative merging resulted in a total of *n*= 343,240 peaks, of which *n* = 67,215 peaks called in >2 tumour regions or the panel of normal were retained. The ChIPseeker v2.14.0 package for R (ref. ^90^) was used in combination with the TxDb.Hsapiens.UCSC.hg38.knownGene package v3.10.0 for R to annotate peaks on the basis of their genomic location. For peaks that were not proximal to known promoter regions (±3,000 bp), overlaps with known enhancer elements reported in the double-elite annotations of the GeneHancer database were examined^91^.

#### Extraction of cut sites in peaks in ATAC peak-calling analysis

Read counts for each peak in the final set were collated using bedtools^92^ using: ‘bedtools coverage -a bed peaks -b bed cut sites -split -counts -sorted’.

#### Purity estimation for ATAC-seq and accounting for CNAs

Clonal variants identified by paired WGS sequencing (clonal variants were those present in all samples from the cancer) were used to estimate sample-specific ATAC-seq purity. First, variants in intervals with identical (clonal) copy-number states (that is, A&B-allele states) and regions of closed chromatin were identified from WGS data. Copy-number values *c*_*i*_ and mutation multiplicity *m*_*i*_ of each variant site *i* were obtained from the WGS data. For a mutation at site *i* covered by *n*_*s*__,__*i*_ reads in sample *s*, the number of reads *k*_*i*_ containing the alternative allele is expected to follow a binomial distribution with the pdf

$$B\left({k}_{i}| {p}_{s,i},{n}_{s,i}\right)=\left(\begin{array}{c}{n}_{s,i}\\ {k}_{i}\end{array}\right){p}_{s,i}^{{k}_{i}}{\left(1-{p}_{s,i}\right)}^{{n}_{s,i}-{k}_{i}}$$

in which the expected success probability *p*_*s*__,__*i*_ is a function of the sample purity ρ_s_^’^ the number of mutated alleles in the tumour cells *m*_*s*,*i*_, the total copy number of the mutated site in the tumour cells *c*_*s*__,*i*_ and the copy number in contaminating normal cells c_n_ = 2

$${p}_{s,i}=\frac{{\rho }_{s}{m}_{s,i}}{{\rho }_{s}{c}_{s,i}+\left(1-{\rho }_{s}\right){c}_{n}}=\frac{{\rho }_{s}{m}_{s,i}}{{\rho }_{s}{c}_{s,i}+2-2{\rho }_{s}}$$

A maximum-likelihood estimate of the sample purity *p*_*s *_was obtained by minimizing the negative-log-likelihood $L({\rho }_{s})=\mathop{\sum }\limits_{i=0}^{N}-\log (B({k}_{i}|{p}_{s,i},{n}_{s,i}))$ across all N mutated sites.

To account for the influence of CNAs on the read counts, the signal observed at a locus should be given by $S={S}_{N}\frac{2\,\left(1-\rho \right)+\pi \rho }{2\left(1-\rho \right)+\psi \rho }$, in which *S*_*N*_ is the signal of the reference allele, $\rho$ is the purity of the sample, $\pi$ is the copy number of the locus, and $\psi$ is the ploidy of the tumour. For pooled samples, we calculate the average of *S* weighted by the total number of reads across samples. Indeed, CNAs were affecting the read depth at the locus (see the figures at 10.6084/m9.figshare.19849789 for details).

However, it is important to consider that, in general, CNAs are causing relatively small changes in the ATAC-seq signals compared to those of bona fide SCAAs. This was demonstrated by the strong correlation of the recurrence number in the model with copy-number adjustment versus the one without. This approach was most relevant in the identification of lost chromatin accessibility in regions with a copy-number gain and gained chromatin accessibility in regions with a copy-number loss.

#### Identification of recurrently altered peaks across patients

Analysis was restricted to samples with purity $\rho$ > 0.4. Peaks proximal (≤1,000 bp) to a transcription start site (TSS; that is, promoters) and those more distant to a TSS (that is, putative enhancers) were considered separately to account for the possibility of differential dispersion. Whereas we relied on proximity for promoters, we used the GeneHancer database for enhancers^91^. An overdispersed Poisson model was fitted to each peak using edgeR v3.30.3 (refs. ^93,94^), per-sample set normalization factors were calculated using the TMMwsp method^95^, a global dispersion estimate was estimated across sets from all cancers and each set of pure glands (per patient) was compared against a large pool of normal tissue ATAC-seq samples. Recurrently altered peaks were identified as those that were significantly altered at a level of *P* ≤ 0.01 in at least 4/26 (that is, 20%) of cases.

#### Identification of associated changes in gene expression

The basic processing of matched RNA-seq data is described in the associated manuscript^38^. A subset of 27,699 peaks that were either adjacent to a known TSS of a gene^96^ or overlapped a previously characterized enhancer element described in the GeneHancer database^91^ were identified. Of these 456/27,699 (≈1.65%) were recurrently altered. Changes in expression of genes associated with these sites were tested for using DESeq2 (ref. ^97^) to compare coefficients of the fitted β-binomial regression model (design: ~Patient, with all normal samples as ‘Normal’) with the contrast argument being a list of vectors containing the significant and non-significant patient sets.

For promoters, a one-tailed hypothesis test was applied by setting the altHypothesis argument to ‘less’ (for closed peaks) or ‘greater’ (for opened peaks). For enhancers, a two-tailed hypothesis test on all associated genes was applied by setting the altHypothesis argument to ‘greaterAbs’. *P* values from all tests were adjusted for multiple-hypothesis testing using FDR method^98^ associations at FDR < 0.1% where reported. For the visualization of gene expression values, the average variance stabilised log-transformed gene expression was calculated across samples of all each cancer and across all normal samples.

#### Identification of subclonal changes in recurrently altered peaks

Subclonality was assessed only for a set of recurrent somatic accessibility changes, comprising recurrent events affecting driver genes and the top 25 most recurrent in each of the 4 categories: gained promoter, lost promoter, gained enhancer and lost enhancer (total of 521 sites assessed).

Our previous analyses recognized that sample purity was highly correlated with tumour piece (regions A–D). To distinguish subclonal chromatin accessibility alterations from variability in ploidy, regression to account for purity was performed. Specifically, a log ratio test from DESeq2 was used to compare a ‘full model’ ~purity + region to a reduced model ~purity. Samples from the same region were used as biological replicates. Events were considered putatively subclonal when the adjusted *P* value was below 0.05 and if the direction of log[fold change] from analysis of matched bulk tissues was correlated with that observed in individual samples. In the case of gained events, subclonal events were filtered out if MACS peak-calling (see above) had not called a peak within 500 bp of the location of the putative gain event (this removed 33 sites). For losses, 5/45 subclonal events were removed as the log[fold change] was in the wrong direction.

For visualization of peaks, coverage per region was calculated 1 kb upstream and 1 kb downstream from the centre of the peak. Coverage was normalized per million reads in peaks and was plotted using functions from GenomicRanges^99^ and Gviz^100^.

#### Prediction of TF-binding sites

The motifmatchr package for R (ref. ^101^), a reimplementation of the C++ library MOODS^102,103^, was used to identify binding sites for all human TF motifs defined in a curated version of the CIS-BP database^104^. The list of predicted binding sites was filtered using a minimum significance value of *P* ≤ 10^−6^, followed by removal of binding sites in centromeric regions and non-autosomal (that is, sex and non-canonical) chromosomes. After this initial filtering, predicted binding sites were split into six distinct groups on the basis of their distance to the next TSS (proximal: *d* ≤ 2,000 bp; close: 2,000 bp < *d* ≤ 10,000 bp; distal: *d* > 10,000 bp) and whether they overlapped with a peak observed in the ATAC-seq data. For a number of TFs, homotypic clustering of binding sites in specific intervals was observed; to account for this, binding sites that were closer than *d* ≤ 1,000 bp to the next predicted binding site of the same TF were removed.

#### Extraction of signal values

For each of the TF sets described above, the counts of insertions around the centre of the TF-binding site (±1,000 bp) as well as the insertion size of the read pair (that is, thedistance to the second nick) for each sample^99^ were tabulated. The insertion sizes (rows) were binned into intervals of 5 bp and divided by the total count of reads with an equivalent size in the entire genome. After this, the background signal was estimated to be theaverage number of insertions 1,000 bp–750 bp from the centre of the TF-binding site per insertion size and subtracted from the counts. The difference between these normalized and background-corrected TF signals in each sample and a pool of normal samples was calculated and integrated across the central region of the TF-binding sites (insertion size [25;120], distances [−100 bp;100 bp]) as a summary statistic. Linear regression analysis was used to identify associations with purity estimates, and in this context, signals were found to correlate with TSS enrichment (TSSe; for both nucleosome-free and all reads). For thisreason, a further term was added to the regression model of each TF to correct for this effect: signal ≈ tsse*tsse_nf_ + purity:patient (where ‘:’ indicates an interaction between two or more variables in the model formula and ‘*’ indicates all the main effects and interactions among the variables that it joins), in which tsse and tsse_nf_ are the differences in TSSe between the sample and the pooled normal samples, and each observation was weighted by the square root of the number of reads in the sample. A second linear model in which a region-specific effect of the purity (signal ≈ tsse*tsse_nf_ + purity:region) was considered was also fitted to the data. For both models, the statistical significance of the purity coefficient was determined. The estimates of the coefficients were also used as a patient-specific summary for subsequent analysis.

#### Cluster analysis

The analysis was focused on the 150 TFs for which a significant association with the tumour cell content (that is, the purity) and TF signal was most frequently observed. With the aim to identify general patterns in these data, a clustering analysis was conducted (hierarchical clustering with Euclidean distance and complete linkage). This method identified three main groups of TFs, each of which was analysed with STRINGdb^105^ to identify significantly overrepresented pathways.

#### Methylation array analysis

A reference normal methylation array dataset was downloaded from ref. ^106^ that included normal tissue sampled adjacent to CRCs that was profiled using the HumanMethylation450 BeadChip array (Illumina).

Here, eight bulk samples from four cases (C516, C518, C560 and C561) were profiled using the MethylationEPIC BeadChip (Infinium) microarray according to the manufacturer’s instructions.

The ChAMP R package pipeline^107^ was used to analyse the methylation bead array data. Probes that had a detection *P* > 0.01 and probes with <3 beads in at least 5% of samples per probe, probes that were on the X or Y chromosome, all probes associated with single nucleotide polymorphisms and all multi-hit probes were removed. Subset-within-array normalization was used to correct for biases resulting from type 1 and type 2 probes on the array. After quality control and normalization, β-values were calculated for further comparison.

To compare the methylation patterns between our samples and the reference normal dataset, the overlapped probes of all samples located distal to the TSS, close to the TSS and proximal to the TSS, both on the ATAC peak and not on the ATAC peak were compared.

#### Processing of RNA-seq

After initial quality control with FastQC (https://github.com/s-andrews/FastQC) and default adapter trimming with Skewer^62^, paired-end reads were aligned to the GRCh38 reference genome and v28 of the Gencode GTF annotation using the STAR two-pass method^108^. Read groups were added with Picard v2.5.0 (http://broadinstitute.github.io/picard). Per-gene read counts were produced with htseq-count, which is incorporated in the STAR pipeline^108^.

#### Filtering of RNA samples

Raw gene counts were first filtered for reads uniquely assigned to non-ribosomal protein-coding genes located on canonical chromosomes (chr1-22, X and Y). If samples had fewer than 5 million of these ‘usable’ reads, they were resequenced to improve coverage. When possible, the same library preparation pool was sent again for sequencing. These ‘top-ups’ proved to be true technical replicates, as the resulting gene expression of the resequenced samples clustered very closely to their original samples on both a sample–sample heatmap and a principal component analysis. It was therefore determined that the FASTQs of these samples could simply be merged at the start of the pipeline. In cases in which resequencing was required but insufficient library remained, a new library was prepared, and the sequencing run that produced the highest read was used in subsequent analysis. For eight samples, the sequencing of the second library contained too few reads to enable downstream analysis. Six of eight samples showed per-gene read counts that were very similar between libraries 1 and 2 (Spearman’s rank correlation between replicates was significantly higher than the mean; Wilcoxon one-way rank test; FDR < 0.01) and so read counts were combined across libraries; the two remaining samples were discarded. Samples were also discarded if matched DNA sequencing revealed a tumour purity of less than 0.1.

#### Gene expression normalization and filtering

The number of non-ribosomal protein-coding genes on the 23 canonical chromosome pairs used for quality control was 19,671. Raw read counts uniquely assigned to these genes were converted into both transcripts per million and variance-stabilizing transformed (vst) counts using DESeq2 (ref. ^97^).

A list of expressed genes (*n* = 11,667) was determined by filtering out genes for which less than 5% of tumour samples had at least 10 transcripts per million. To concentrate on tumour epithelial cell gene expression, genes were further filtered out if they negatively correlated with purity as estimated from matched DNA-sequencing data. Specifically, for the 157 tumour samples that had matched DNA sequencing and therefore accurate purity estimates, a linear mixed-effects model of exp(vst) ≈ Purity + (1|Patient) was compared using a chi-squared test to exp ≈ (1|Patient). Genes that had a negative coefficient for purity in the first model and an FDR-adjusted *P* value less than 0.05, suggesting that purity significantly affected the expression, were filtered out. This led to a filtered list of 11,401 expressed genes.

#### Mutational signature analysis

Mutational signature analysis was performed with SparseSignatures^51^. This method uses LASSO regularization^109^ to reduce noise in the signatures, controlled by a regularization parameter lambda (*λ*). It implements a procedure based on bi-cross-validation^110^ to select the best values for both the regularization parameter *λ* and the number of signatures. Deconvolution using a maximum of 10 signatures was performed and values of *λ* of 0.000, 0.025, 0.050 and 0.100 were tested. Optimal parameters were selected on the basis of the median bi-cross-validation error estimated over 1,000 iterations, resulting in an optimal estimate with minimum cross-validation median error when 6 signatures were fitted and *λ* = 0.025. A second analysis with SigProfiler^111^, with default parameters and a total of 1,000 iterations, confirmed the existence of these signatures.

Signature-based clustering was performed considering the six-signature solution by SparseSignatures; the signatures exposure matrix given as an output by the tool was used to compute the pairwise similarity matrix for each patient as 1 minus the cosine similarity of their exposures. Clustering was then performed on the similarity matrix by *k*-means with six clusters explaining all of the variance. Although from a statistical perspective clusters C3 and C4 are defined by a small number of samples (and explain 3% and 4% of the variance, respectively), from the biological perspective, we have evidence that in these patients the distribution of mutations resembles very different signatures and mutational processes (Supplementary Fig. 15A).

Mutational signature exposures were also analysed across epigenetic regions. Mutations were first grouped as clonal or subclonal across the whole genome and then in different genomic regions (as described above). Signature activities in each region were estimated by jackknife sampling^112^. Specifically, data from each patient were partitioned on the basis of their clusters as defined above, and repeated jackknife sampling was performed 100 times independently for each of the 3 clusters (including a random sample of 90% of the tissue samples each time). For each iteration, the mutations in each genomic region were used to compute a data matrix normalized against the trinucleotide content (across the 96 channels) in the whole genome versus region-specific counts, and signature assignments were then performed on the normalized data by LASSO^51,109^. Finally, relative signature activities estimated over the 100 jackknife samples were normalized on the basis of the total size of each region. Moreover, as clusters C3 and C4 represent rare and very distinct mutational patterns, we excluded these samples from the estimation of mutational processes in the epigenetic regions by jackknife, and instead we focused on MSS (cluster 1) versus MSI (clusters 2 and 5) tumour, as the samples in clusters C3 and C4 would probably have biased the jackknife estimation for these two groups.

### Reporting summary

Further information on research design is available in the Nature Research Reporting Summary linked to this article.

## Online content

Any methods, additional references, Nature Research reporting summaries, source data, extended data, supplementary information, acknowledgements, peer review information; details of author contributions and competing interests; and statements of data and code availability are available at 10.1038/s41586-022-05202-1.

### Supplementary information

Supplementary Information This file contains Supplementary Figs. 1–19.

Reporting Summary

Supplementary Table 1 Clinicopathological annotation of the analysed tissue samples.

Supplementary Table 2 Annotation of ATAC-seq-profiled tissue samples.

Supplementary Table 3 Annotation of WGS-profiled tissue samples.

Supplementary Table 4 Annotation of RNA-seq profiled tissue samples.

Supplementary Table 5 List of chromatin modifier genes.

Supplementary Table 6 List of driver genes.

Supplementary Table 7 Summary of the recurrence of SCAAs and their associated gene expression changes.

## Data Availability

Gene expression data, somatic mutation calls (VCF files from Mutect2 plus Platypus), copy-number calls (Sequenza and QDNAseq), the fraction of mutated microsatellites (MSIsensor), ATAC-seq insertion counts and allele counts of somatic SNVs in all sample types are available on Mendeley (10.17632/7wx3chtsxx.2). Other figures have been deposited in Figshare (10.6084/m9.figshare.c.6011476.v1). Sequence data have been deposited at the European Genome-phenome Archive, which is hosted by the European Bioinformatics Institute and the Centre for Genomic Regulation, under accession number EGAS00001005230. Further information about the European Genome-phenome Archive can be found at https://ega-archive.org. Access to these data is restricted and subject to application.

## Code availability

Complete scripts to replicate all bioinformatic analysis are available at https://github.com/sottorivalab/EPICC2021_data_analysis. Further exploration of the ATAC-seq data shown in Fig. 3 can be carried out using a Shiny-App accessible at https://theide.shinyapps.io/EPICC_shiny_app/. A local version of the app and the associated source code can be obtained from https://github.com/sottorivalab/EPICC_shiny_app.
