# Supplementary material for: The co-evolution of the genome and epigenome in colorectal cancer
Source: Nature. 2022 Oct 26;611(7937):733–43. doi: 10.1038/s41586-022-05202-1 (PMC9684080; doi:10.1038/s41586-022-05202-1)
Supplement: Supplementary file 1 — This file contains Supplementary Figs. 1–19. [file 41586_2022_5202_MOESM1_ESM.pdf]

nature portfolio

https://doi.org/10.1038/s41586-022-05202-1

Supplementary information

The co-evolution of the genome and epigenome in colorectal cancer

In the format provided by the authors and unedited

Supplementary Figures

The co-evolution of the genome and epigenome in colorectal cancer

Timon Heide[1,2,*], Jacob Househam[1,3], George D Cresswell[1], Inmaculada Spiter[1], Claire Lynn[1], Maximilian Mossner[1,3], Chris Kimberley[3], Javier Fernandez-Mateos[1], Bingjie Chen[1], Luis Zapata[1], Chela James[1], Iros Barozzi[4,5], Ketevan Chkhaidze[1], Daniel Nichol[1], Vinaya Gunasri[1,3], Alison Berner[3], Melissa Schmidt[3], Eszter Lakatos[1,3], Ann-Marie Baker[1,3], Helena Costa[6], Miriam Mitchinson[6], Rocco Piazza[7], Marnix Jansen[6], Giulio Caravagna[1,8], Daniele Ramazzotti[7], Darryl Shibata[9], John Bridgewater[10], Manuel Rodriguez-Justo[6], Luca Magnani[4], Trevor A Graham[1,3,†], and Andrea Sottoriva[1,2,†]

[1]Centre for Evolution and Cancer, The Institute of Cancer Research, London, UK
[2]Computational Biology Research Centre, Human Technopole, Milan, Italy
[3]Evolution and Cancer Lab, Centre for Genomics and Computational Biology, Barts Cancer Institute, Queen Mary University of London, London, UK
[4]Department of Surgery and Cancer, Imperial College London, London, UK
[5]Centre for Cancer Research, Medical University of Vienna, Vienna, Austria
[6]Department of Pathology, UCL Cancer Institute, University College London, London, UK
[7]Department of Medicine and Surgery, University of Milano-Bicocca, Milan, Italy
[8]Department of Mathematics and Geosciences, University of Triest, Triest, Italy
[9]Department of Pathology, University of Southern California Keck School of Medicine, Los Angeles, CA, 90033, USA
[10]UCL Cancer Institute, University College London, London, UK
[*]equal contribution
[†]Correspondence to: trevor.graham@icr.ac.uk and andrea.sottoriva@fht.org

Contents

1 Tissue collection **3**

2 Assay QC **4**

3 CNA analysis **5**

4 SNV analysis **6**

5 ATAC-seq analysis **7**

6 Transcription factor analysis **9**

7 Epigenetic & genetic distances **12**

8 Mutational signatures **13**

List of Supplementary Figures

S1 Colectomy specimen collection images 3
S2 Correlation between average gene expression in TCGA and our normal samples 4
S3 Copy number alteration profiles 5
S4 Mutations in chromatin modifier genes for all samples 6
S5 Peak tracks for additional examples 7
S6 Comparison of peak coverage in our cohort with those of TCGA and ENCODE CRC ATAC-seq data. 7
S7 SCAA burden of adenomas vs carcinomas 8
S8 SCAAs identified in individual normal glands 8
S9 Gene expression of TFs from cluster 1 of heatmap in Figure 4A 9
S10 Methylation levels of CIMP markers of cancers and normal 10
S11 Demethylation in reactivated TF binding sites 10
S12 Methylation of cancer vs normal for housekeeping and normal CRCs methylated genes 11
S13 Heritability of chromatin accessibility 12
S14 Coefficients of the PERMANOVA test for regional effect on epigenetic distances . . . 13
S15 Mutational signature discovery with SparseSignatures 13
S16 Mutational signature deconvolution with SigProfiler 14
S17 Accumulation of different mutational signatures in distinct epigenetic regions 14
S18 Predicted versus observed mutational signatures that cause gain and loss of CTCF . . 15
S19 Proportion of each signature contributing to mutations affecting CTCF binding 15

1 Tissue collection

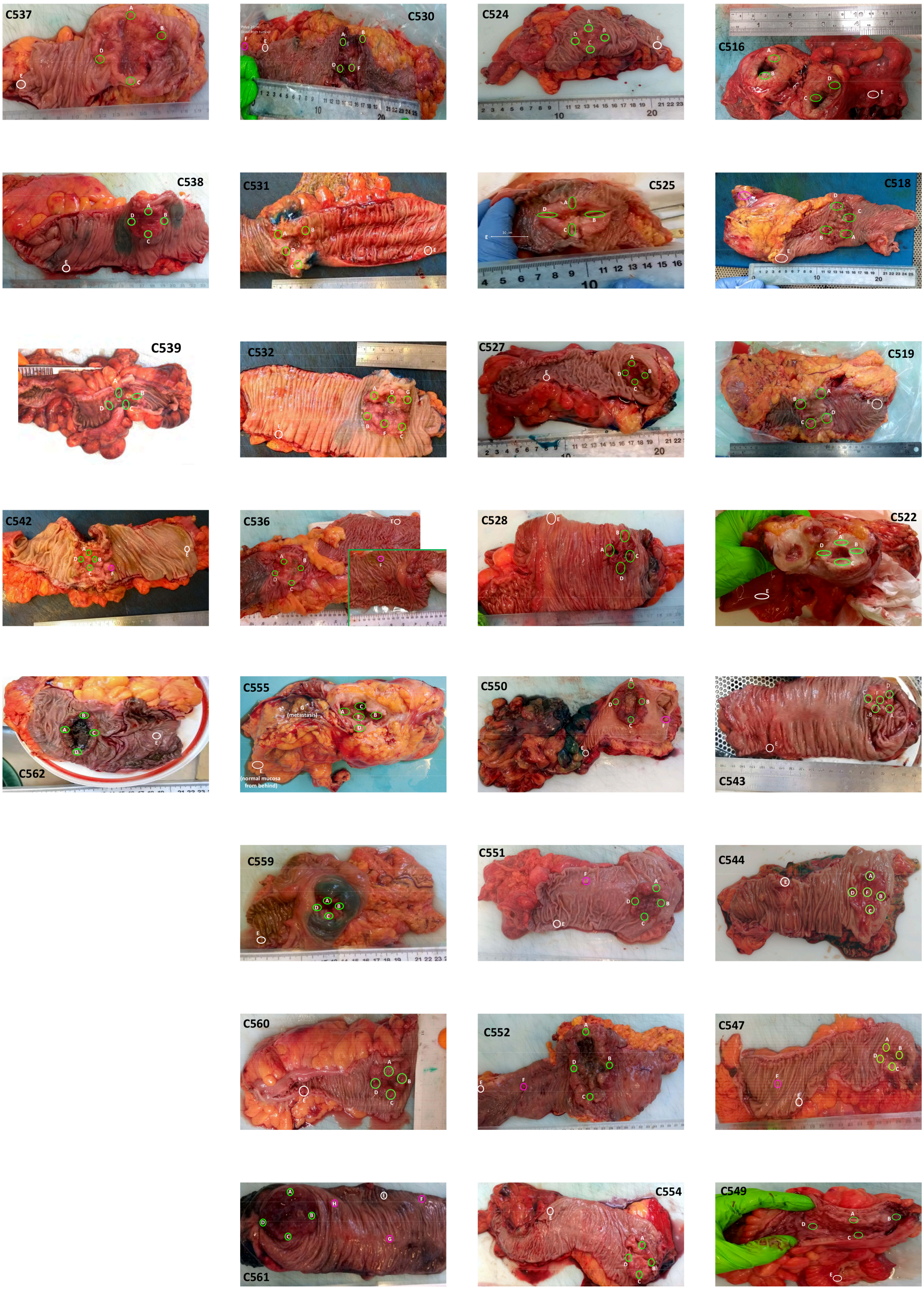

Supplementary Figure S1: Colectomy specimen collection images. Resection specimens were collected from UCLH and sampled with the supervision of a pathologist. Spatial information on different regional samples was retained and indicated in the images. A, B, C, D are cancer regions. E is distant normal epithelium. Eventual concomitant adenomas are reported as F, G, H, etc.

2 Assay QC

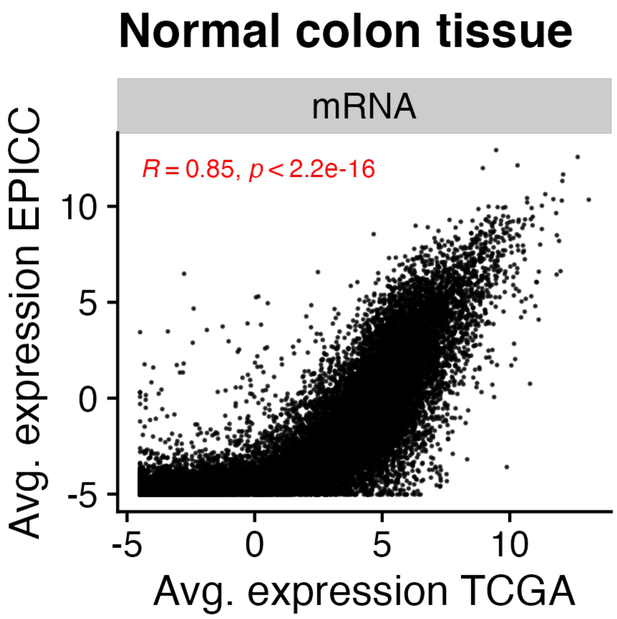

Supplementary Figure S2: Correlation between average gene expression in TCGA normal colon samples ($N = 41$) and our normal samples ($N = 18$). The Pearson correlation coefficient (R) and the associated p-value are shown in red.

3 CNA analysis

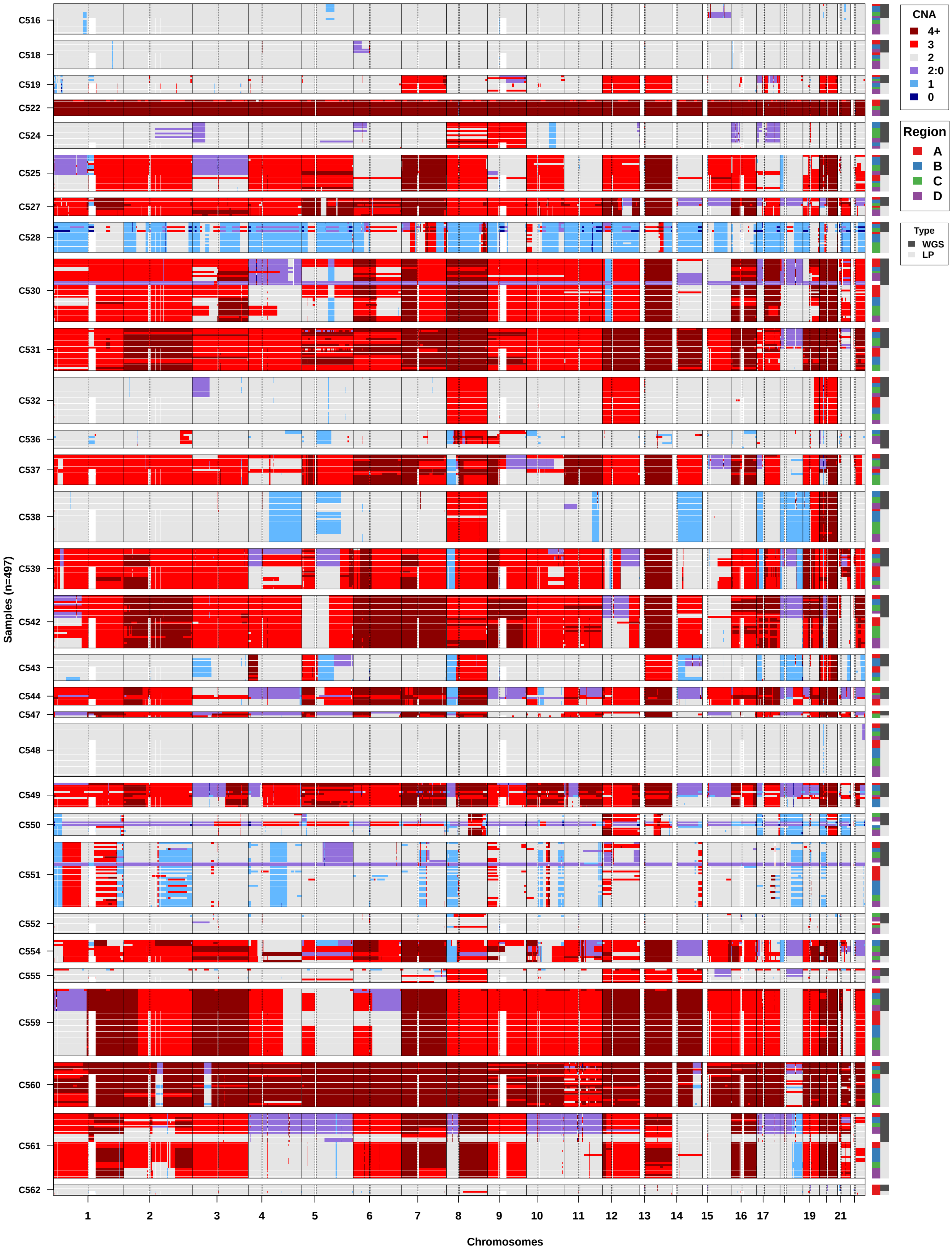

Supplementary Figure S3: Copy number alteration profiles. We estimated absolute copy number alterations for each sample in each patient, both for deep WGS and low-pass WGS.

4 SNV analysis

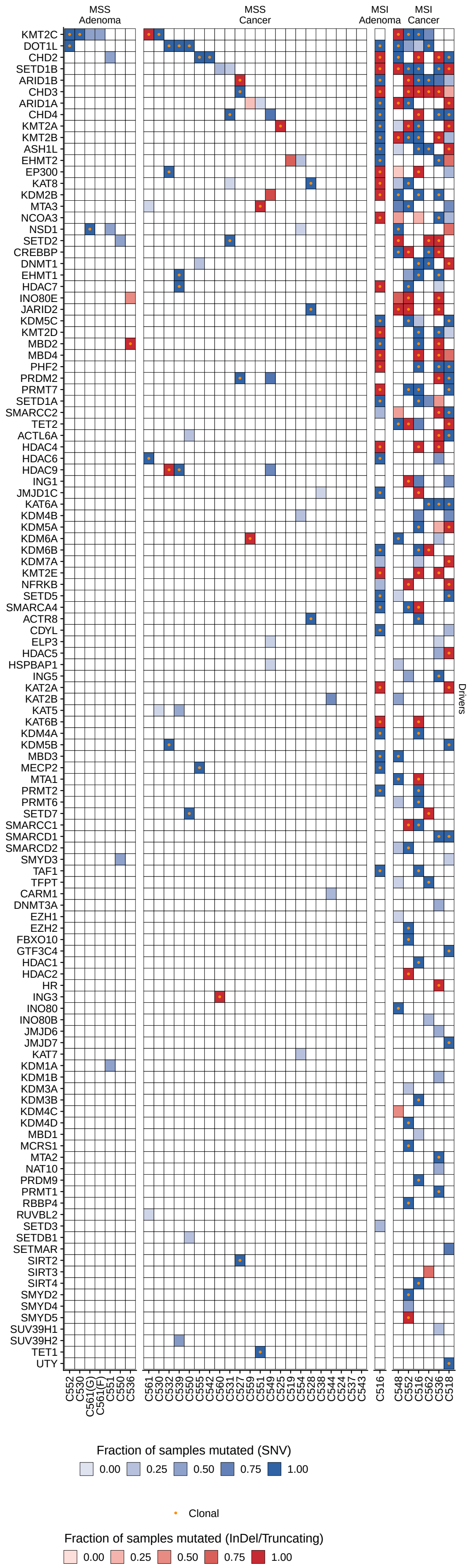

Supplementary Figure S4: Mutations in chromatin modifier genes for all samples.

5 ATAC-seq analysis

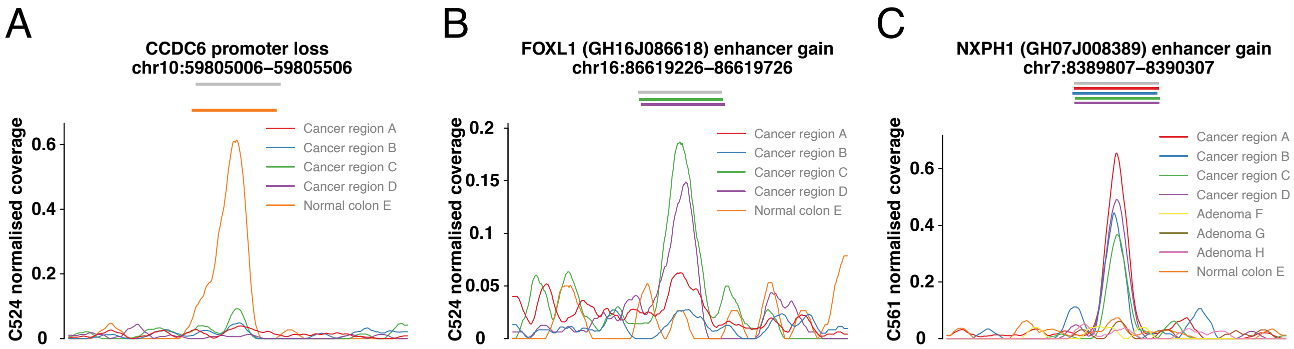

Supplementary Figure S5: Peak tracks for additional examples. (A) Recurrent promoter loss of accessibility of colorectal cancer driver CCDC6, example from C524. (B) FOXL1 enhancer gain of accessibility was found in regions B and C of C524 but not in other regions. (C) Example of somatic peak in NXPH1 enhancer gain found in the cancer but not in the concomitant adenomas of C561. All heterogeneous peaks were identified accounting for purity differences.

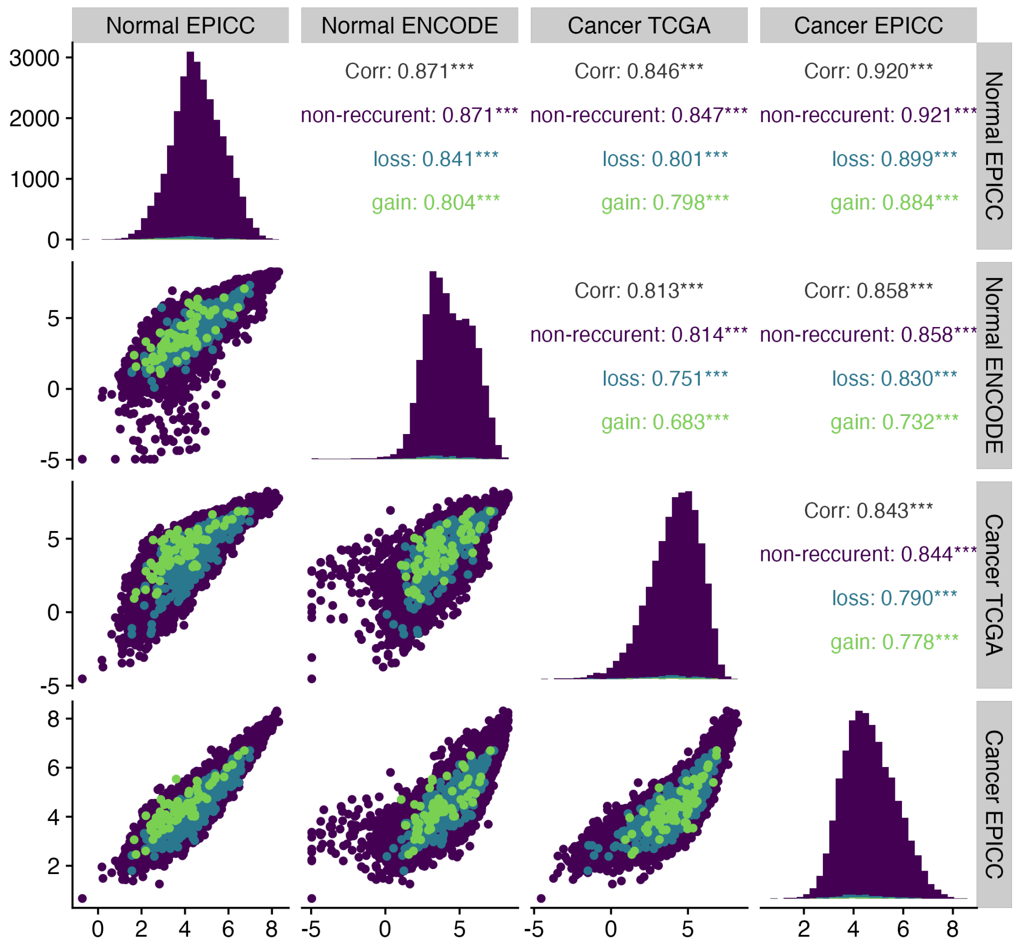

Supplementary Figure S6: Comparison of peak coverage in our cohort with those of from reanalysed TCGA (colorectal cancer) and ENCODE (normal colon) ATAC-seq data.

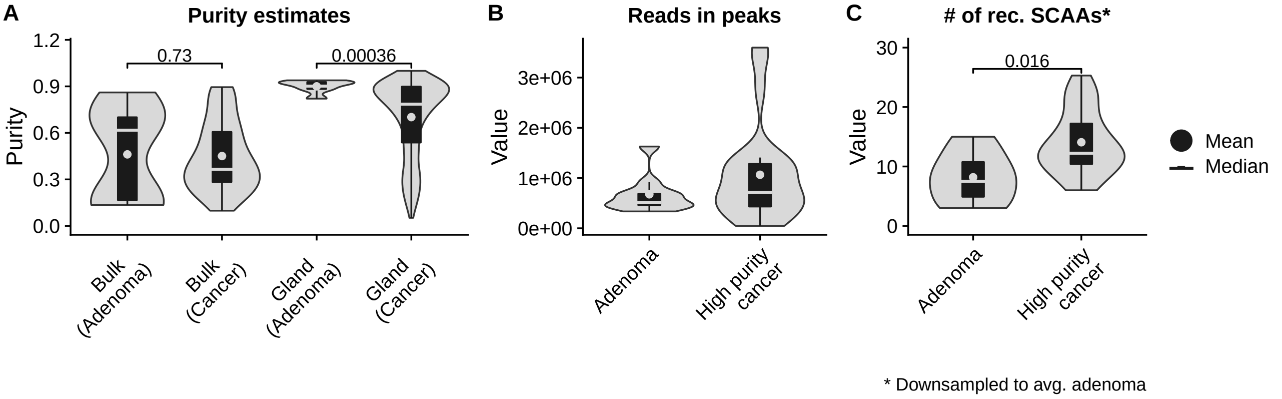

Supplementary Figure S7: SCAA burden of adenomas vs carcinomas (A) Purity of adenomas and carcinomas are comparable, excluding the differences in chromatin accessibility are due to cellularity. Shown p-values indicate the significance of a two-sided t-test (Number of samples: 13 adenoma bulks, 10 carcinoma bulks, 10 adenoma glands and 440 carcinoma glands). (B) Coverage differences are appreciable between cancers and adenomas, however when adjusted for number of reads in peaks (Number of tissues: 8 Adenoma, 24 Carcinoma) (C) it is the case that SCAA burden is significantly higher (two-sided t-test) in carcinomas after correcting for the difference in the number of reads by subsampling the carcinoma data (Number of tissues: 8 Adenoma, 24 Carcinoma).

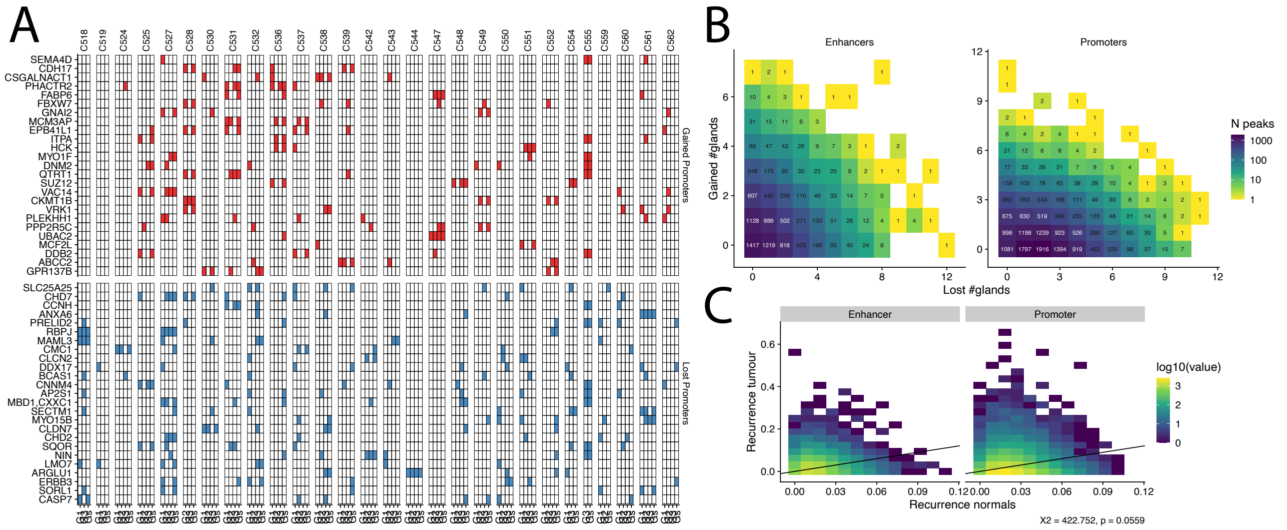

Supplementary Figure S8: SCAAs identified in individual normal glands. (A) Heatmap of recurrent losses and gains promoter SCAAs identified in normal glands. This figure is equivalent to that shown in Figure 3 of the main manuscript. The last column of each patient shows if reads showed significantly differentially accessibility in a pool of all normal glands of patient. (B) Shows the distribution of losses and gains for all peaks. (C) Shows the lack of correlation of the recurrence of SCAAs in normal glands and the cancers.

6 Transcription factor analysis

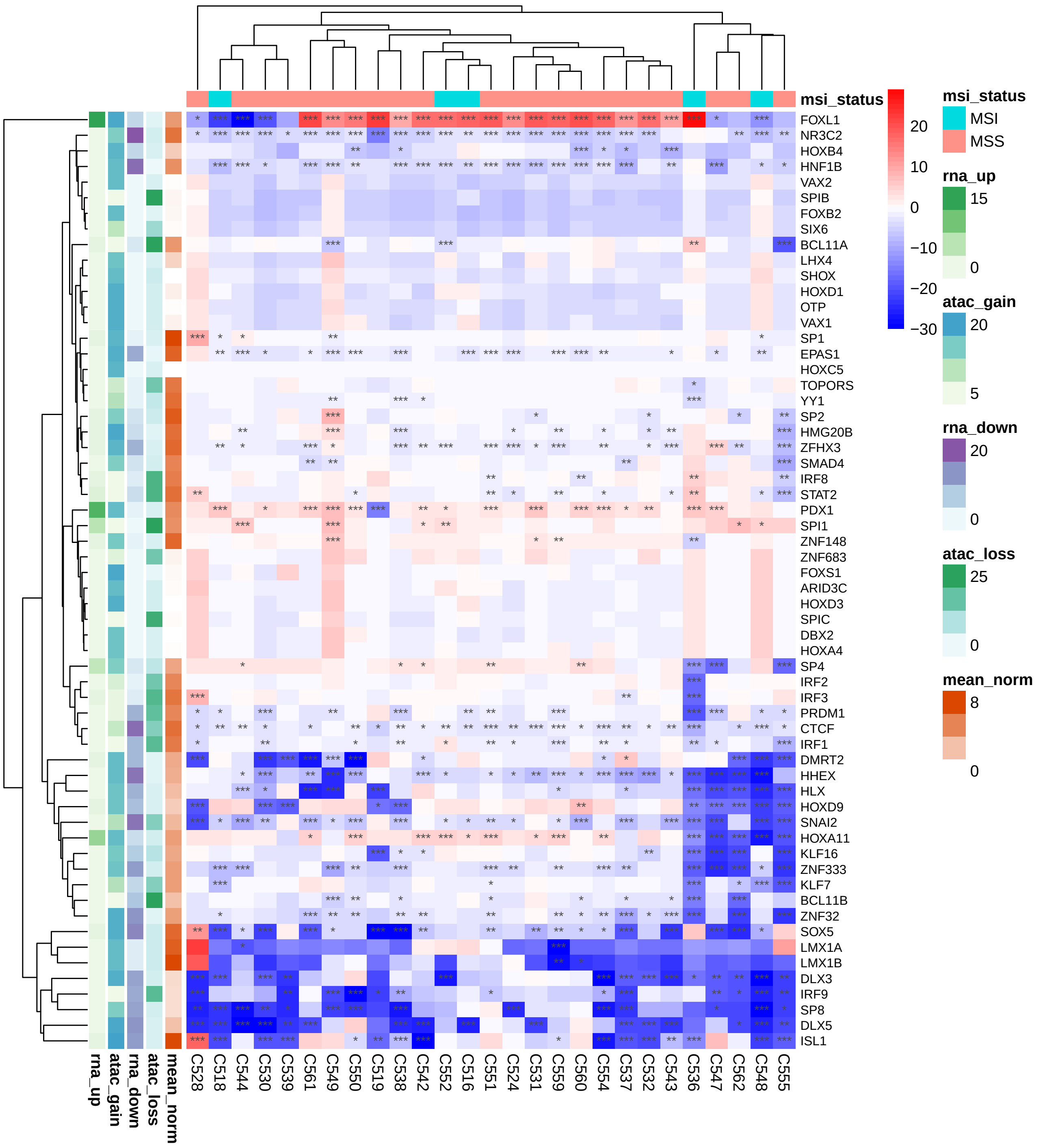

Supplementary Figure S9: Gene expression of TFs from cluster 1 of heatmap in Figure 4A.

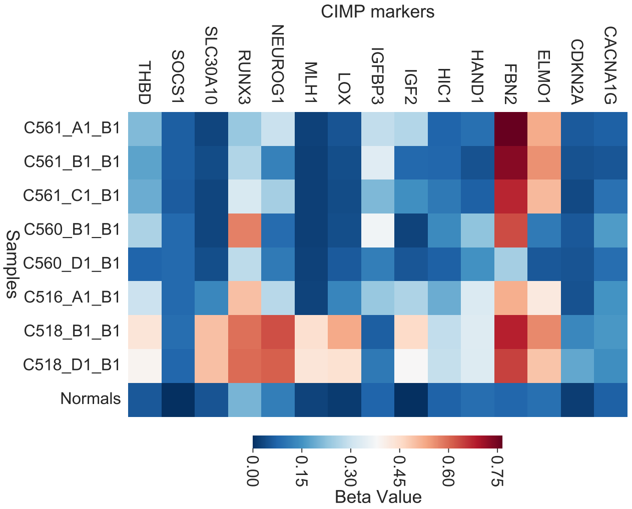

Supplementary Figure S10: Methylation levels of CIMP (CpG Island Methylation Phenotype) markers of cancers and normal.

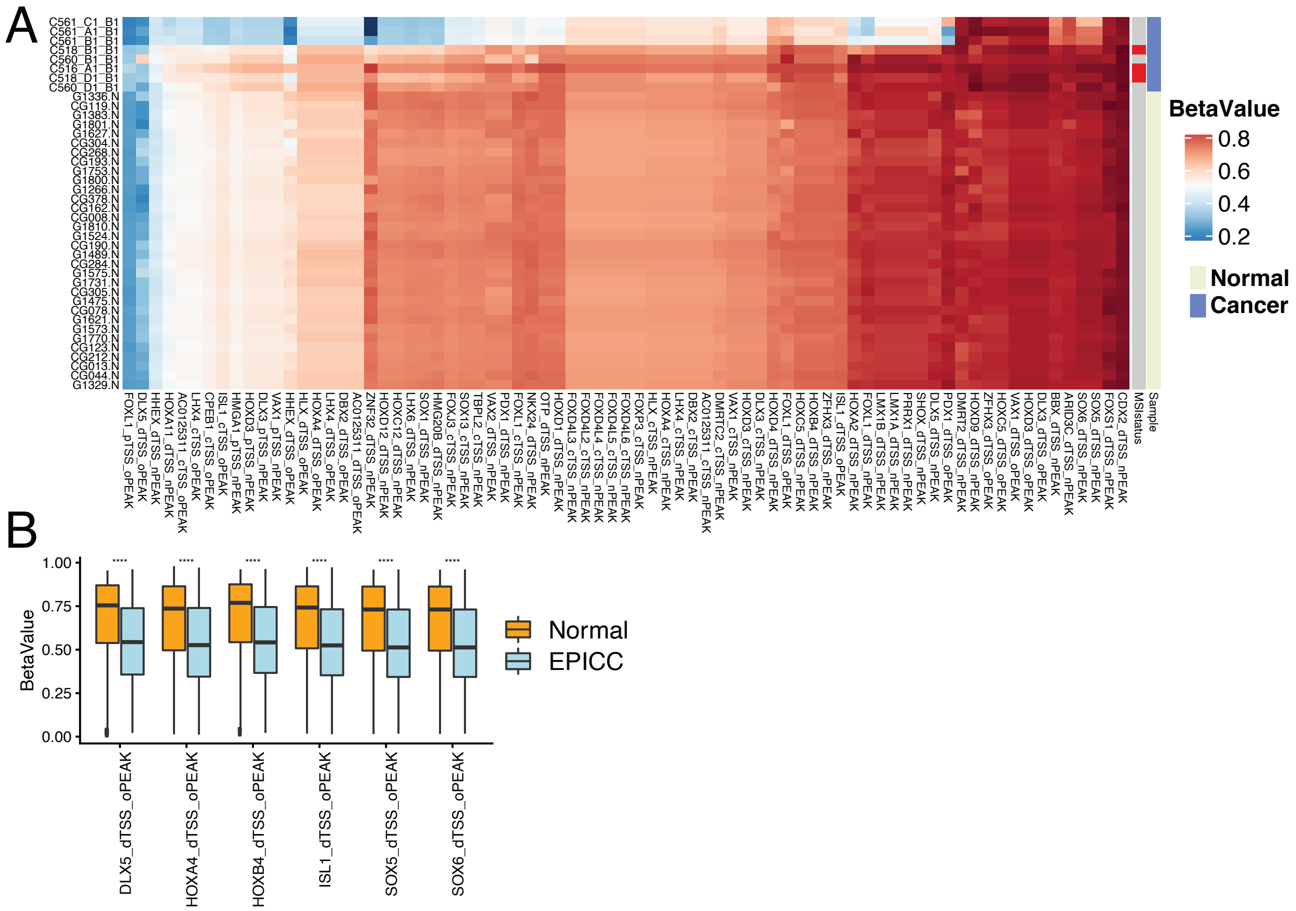

Supplementary Figure S11: Demethylation in reactivated TF binding sites. (A) We selected genomic regions in cluster 3 (enriched in developmental genes like SOX and HOX families) and verified their methylation status with CpG methylation arrays in EPICC samples versus normal. (B) In particular regions corresponding to binding sites of DLX5, HOXA4, HOXB4, ISL1, SOX5 and SOX6 showed decreased methylation in cancer vs normal. The lower and upper hinges of the boxes show the first and third quartiles, the whiskers extend to the most extrem values up to 1.5 inter quartile ranges from the whisker and values outside of this range are shown as individual points. The black horizontal lines within the box shows the median values. Difference between groups was tested using a wilcox test and showed highly significant p-values ($p < 10^{-12}$) for all comparisons. The number of compared CpG probes were 873 for DLX5_dTSS_oPEAK, 2620 for HOXA4_dTSS_oPEAK, 633 for HOXB4_dTSS_oPEAK, 2268 for ISL1_dTSS_oPEAK and 1115 for SOX5_dTSS_oPEAK/SOX6_dTSS_oPEAK.

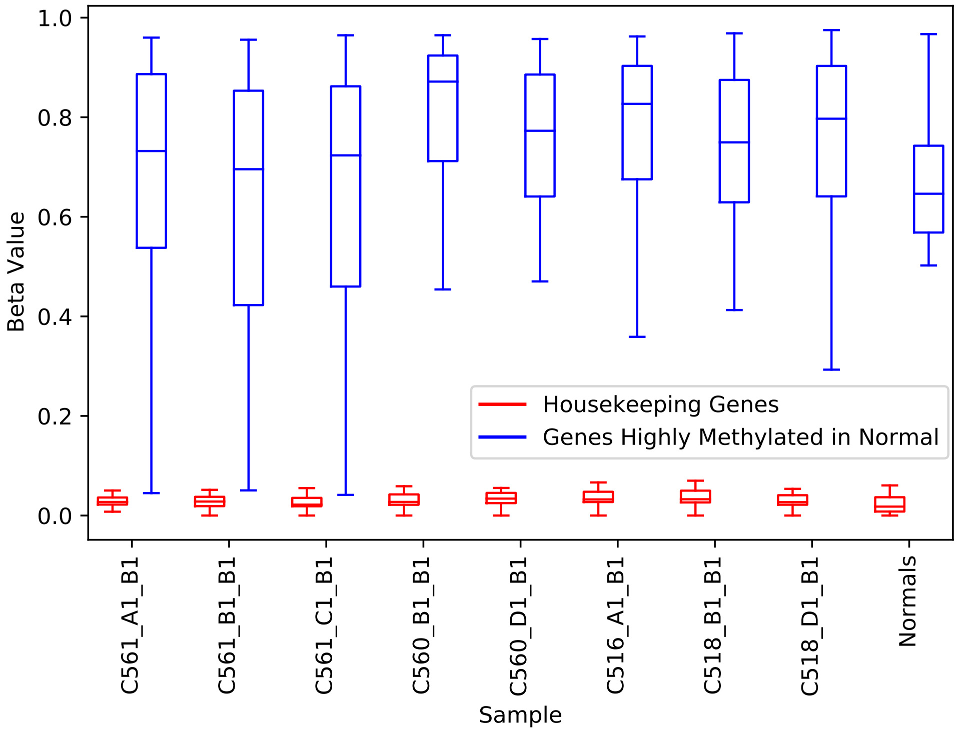

Supplementary Figure S12: Methylation levels of cancer vs normal for housekeeping genes and genes that are usually methylated in normal. These results exclude a global hypomethylation pattern in the cancers. The lower and upper hinges of the boxes show the first and third quartiles, the horizontal lines within the box shows the median value and the whiskers extend to the minimum and maximum value.

7 Epigenetic & genetic distances

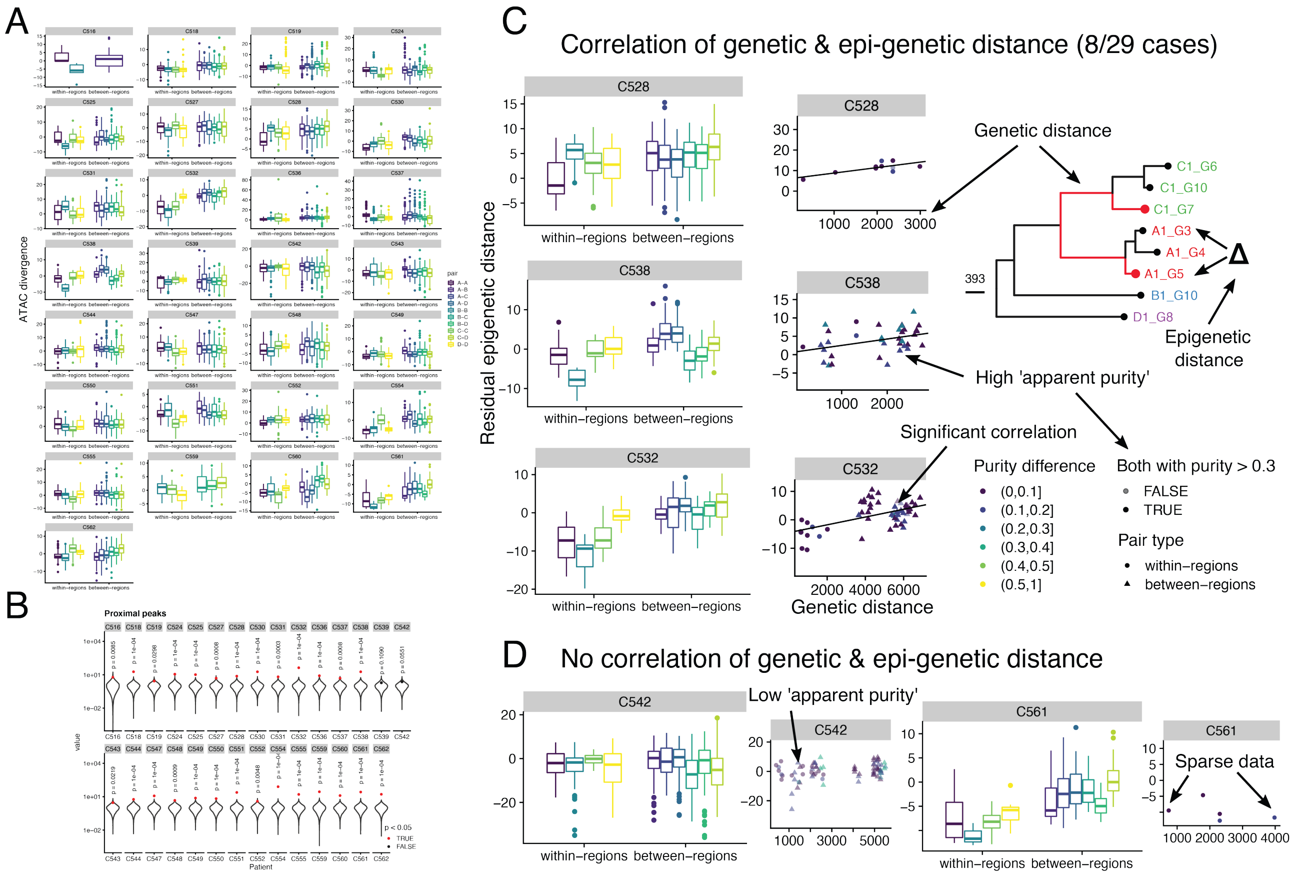

Supplementary Figure S13: Heritability of chromatin accessibility. (A) We compared ATAC distance (euclidean on promoter peaks) between glands from the same region (within-region) and glands of different regions (between-regions) to evaluate divergence of chromatin against space and genetic distance. (B) For the large majority of patients within-region ATAC distance is significantly lower than between region, indicating heritability of the chromatin that follows the spatial and phylogenetic structure of the tumour. Here we plot the F statistics of the ANOVA model on TSSe, number of reads, and region. (C) The distances between and within regions (left) and correlations with the genetic distance (right). The lower and upper hinges of the boxes show the first and third quartiles, the whiskers extend to the most extrem values up to 1.5 inter quartile ranges from the whisker and values outside of this range are shown as individual points. The horizontal lines within the box shows the median values. (D) Cases in which no correlation with the genetic distances existed data were often from low purity samples or sparse. The lower and upper hinges of the boxes show the first and third quartiles, the whiskers extend to the most extrem values up to 1.5 inter quartile ranges from the whisker and values outside of this range are shown as individual points. The horizontal lines within the box shows the median values. The number of profiled samples can be found in Supplementary Table 2 & 3.

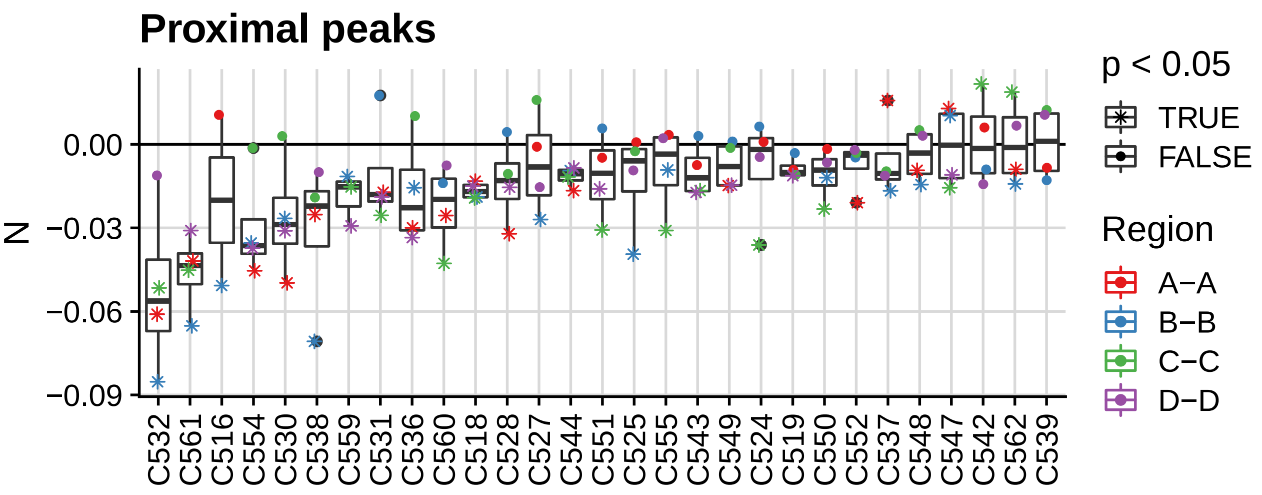

Supplementary Figure S14: Coefficients of the PERMANOVA test for regional effect on epigenetic distances. The lower and upper hinges of the boxes show the first and third quartiles, the whiskers extend to the most extrem values up to 1.5 inter quartile ranges from the whisker and values outside of this range are shown as individual points. The number of ATAC-seq profiled regions of each case can be found in Supplementary Table 2 & 3.

8 Mutational signatures

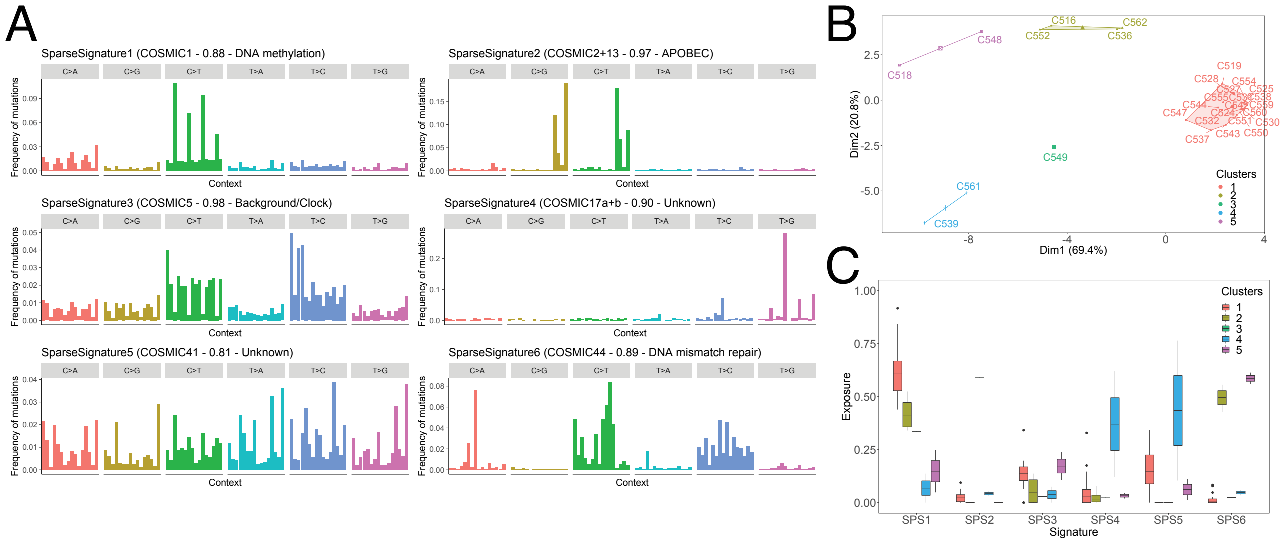

Supplementary Figure S15: Mutational signature discovery with SparseSignatures. (A) Mutational signature discovery with sparse signatures identified 6 signatures in our cohort. (B) Principal Component Analysis divided the patients into 5 clusters depending on contribution from each signature. (C) Signature activity varied between clusters. The lower and upper hinges of the boxes show the first and third quartiles, the whiskers extend to the most extrem values up to 1.5 inter quartile ranges from the whisker and values outside of this range are shown as individual points. The horizontal lines within the box shows the median values. The number of tumours per cluster are 21, 4, 1, 2 and 2.

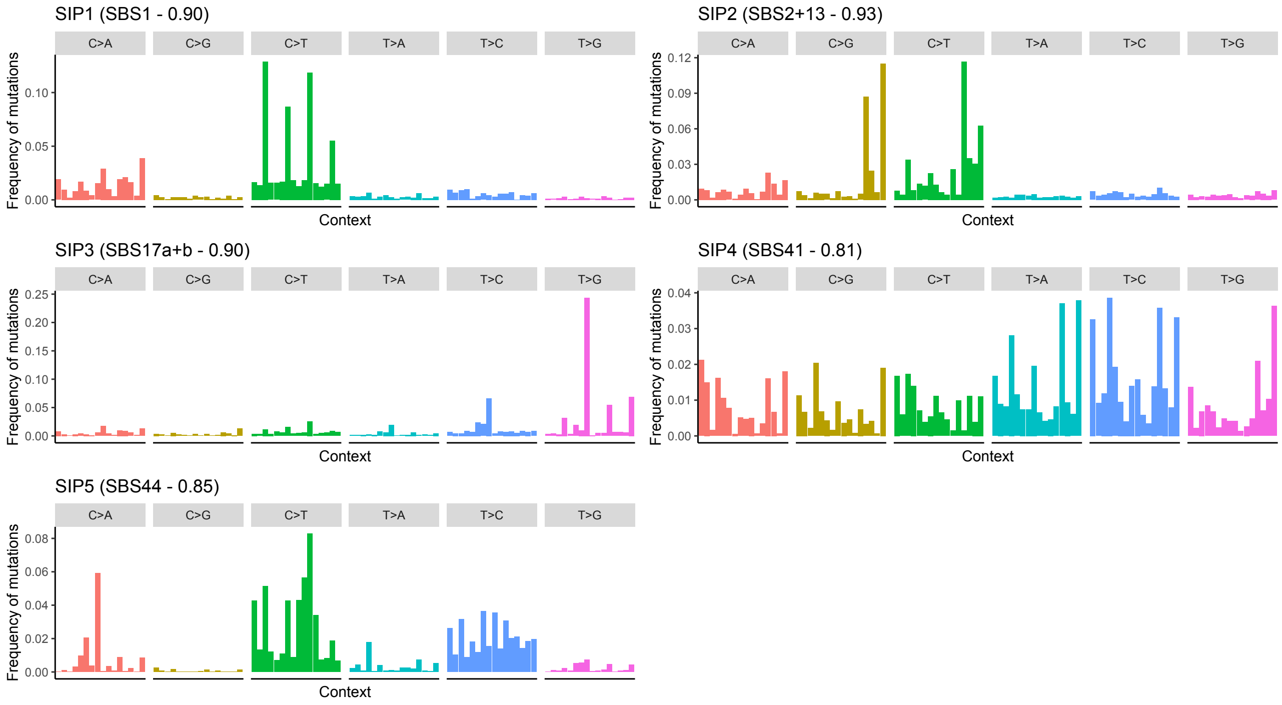

Supplementary Figure S16: Mutational signature deconvolution with SigProfiler.

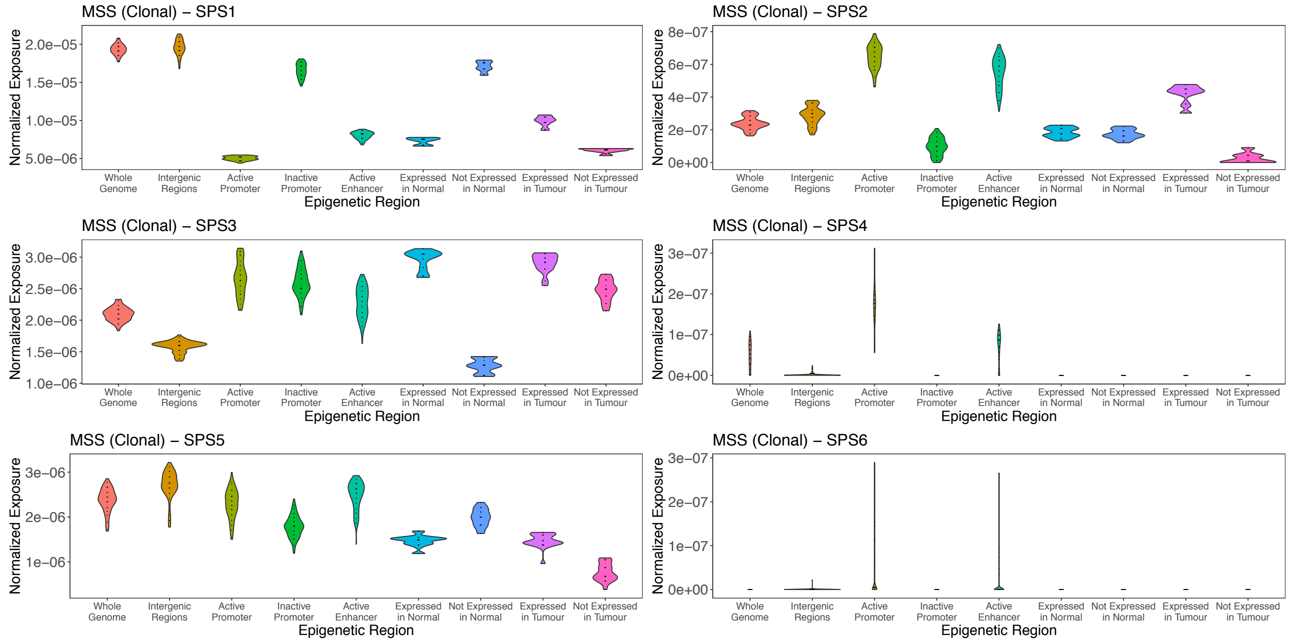

Supplementary Figure S17: Accumulation of different mutational signatures in distinct epigenetic regions.

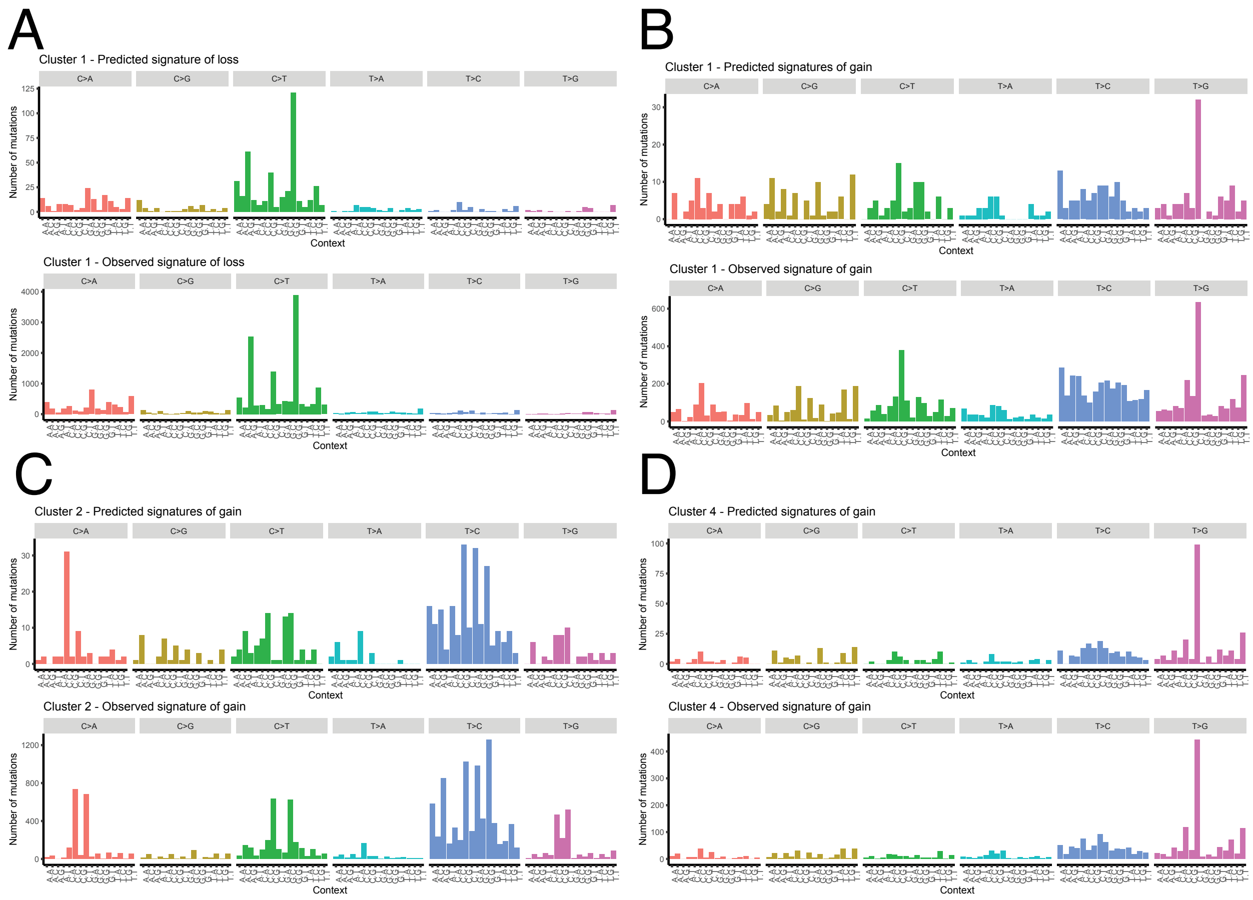

Supplementary Figure S18: Predicted versus observed mutational signatures that cause gain and loss of CTCF.

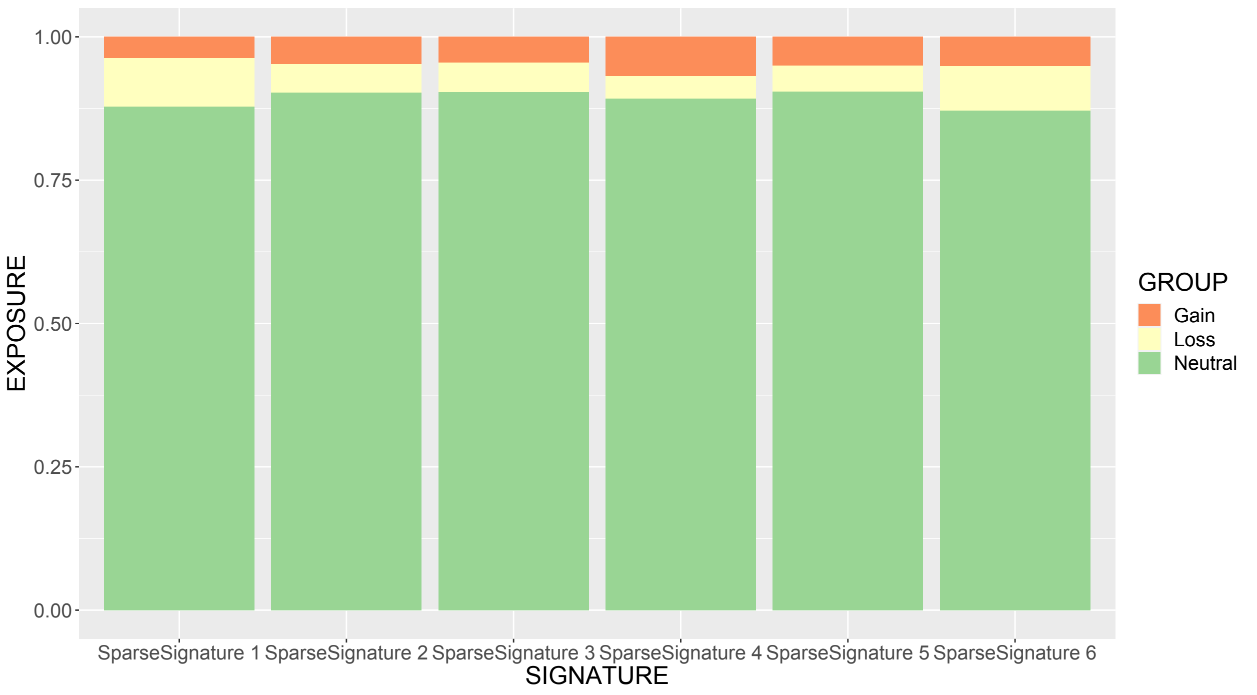

Supplementary Figure S19: Proportion of each signature contributing to mutations affecting CTCF binding.
