# Supplementary material for: The co-evolution of the genome and epigenome in colorectal cancer
Source: Nature. 2022 Oct 26;611(7937):733–43. doi: 10.1038/s41586-022-05202-1 (PMC9684080; doi:10.1038/s41586-022-05202-1)
Supplement: Supplementary file 2 — Reporting Summary [file 41586_2022_5202_MOESM2_ESM.pdf]

nature portfolio

Corresponding author(s): Trevor A Graham, Andrea Sottoriva

Last updated by author(s): Mar 24, 2022

Reporting Summary

Nature Portfolio wishes to improve the reproducibility of the work that we publish. This form provides structure for consistency and transparency in reporting. For further information on Nature Portfolio policies, see our Editorial Policies and the Editorial Policy Checklist.

Statistics

For all statistical analyses, confirm that the following items are present in the figure legend, table legend, main text, or Methods section.

n/a	Confirmed	
☐	☒	The exact sample size (*n*) for each experimental group/condition, given as a discrete number and unit of measurement
☐	☒	A statement on whether measurements were taken from distinct samples or whether the same sample was measured repeatedly
☐	☒	The statistical test(s) used AND whether they are one- or two-sided<br>*Only common tests should be described solely by name; describe more complex techniques in the Methods section.*
☒	☐	A description of all covariates tested
☐	☒	A description of any assumptions or corrections, such as tests of normality and adjustment for multiple comparisons
☐	☒	A full description of the statistical parameters including central tendency (e.g. means) or other basic estimates (e.g. regression coefficient) AND variation (e.g. standard deviation) or associated estimates of uncertainty (e.g. confidence intervals)
☐	☒	For null hypothesis testing, the test statistic (e.g. *F*, *t*, *r*) with confidence intervals, effect sizes, degrees of freedom and *P* value noted<br>*Give P values as exact values whenever suitable.*
☒	☐	For Bayesian analysis, information on the choice of priors and Markov chain Monte Carlo settings
☒	☐	For hierarchical and complex designs, identification of the appropriate level for tests and full reporting of outcomes
☒	☐	Estimates of effect sizes (e.g. Cohen's *d*, Pearson's *r*), indicating how they were calculated

Our web collection on statistics for biologists contains articles on many of the points above.

Software and code

Policy information about availability of computer code

Data collection	see Materials and Methods section of the manuscript
Data analysis	Complete scripts to replicate all bioinformatic analysis and perform simulations and inference are available at: https://github.com/sottorivalab/EPICC2021_data_analysis. Further exploration of the ATAC-seq data shown in Figure 3 can be done using a Shiny-App accessible at https://theide.shinyapps.io/EPICC_shiny_app/.

For manuscripts utilizing custom algorithms or software that are central to the research but not yet described in published literature, software must be made available to editors and reviewers. We strongly encourage code deposition in a community repository (e.g. GitHub). See the Nature Portfolio guidelines for submitting code & software for further information.

Data

Policy information about availability of data

All manuscripts must include a data availability statement. This statement should provide the following information, where applicable:

- Accession codes, unique identifiers, or web links for publicly available datasets
- A description of any restrictions on data availability
- For clinical datasets or third party data, please ensure that the statement adheres to our policy

Analysed data are available on Mendeley: https://data.mendeley.com/datasets/dvv6kf856g/2. Sequence data have been deposited at the European Genome-phenome Archive (EGA), which is hosted by the EBI and the CRG, under accession number EGAS00001005230. Further information about EGA can be found on https://ega-archive.org.

Field-specific reporting

Please select the one below that is the best fit for your research. If you are not sure, read the appropriate sections before making your selection.

☒ Life sciences ☐ Behavioural & social sciences ☐ Ecological, evolutionary & environmental sciences

For a reference copy of the document with all sections, see nature.com/documents/nr-reporting-summary-flat.pdf

Life sciences study design

All studies must disclose on these points even when the disclosure is negative.

Sample size	The number of WGS profiled samples and regions were motivated by previous power analysis. ATAC-seq and RNA-seq were conducted on as many samples as feasible. The total number of cases (N=30) were chosen with the goal to identify the most recurrent alterations.
Data exclusions	RNA-seq data were filtered based on matched WGS purity (<0.05), <5M reads after top-ups. ATAC-seq data with a apparent purity below 40% were not used for the identification of recurrent SCAAs. WGS data with a purity below 20% were excluded from the analysis.
Replication	Different tumours serve as biological replicates representative of CRCs.
Randomization	For the analysis of ATAC-seq data the number of reads, TSS enrichment, copy-number states and sample purity were adjusted. For the analysis of whole-genome sequencing data purity and copy-number alterations were taken into account as covariates.
Blinding	All investigators were blinded to patient data related to outcome, gender, etc.

Reporting for specific materials, systems and methods

We require information from authors about some types of materials, experimental systems and methods used in many studies. Here, indicate whether each material, system or method listed is relevant to your study. If you are not sure if a list item applies to your research, read the appropriate section before selecting a response.

Materials & experimental systems

n/a	Involved in the study	
☒	☐	Antibodies
☒	☐	Eukaryotic cell lines
☒	☐	Palaeontology and archaeology
☒	☐	Animals and other organisms
☐	☒	Human research participants
☒	☐	Clinical data
☒	☐	Dual use research of concern

Methods

n/a	Involved in the study	
☒	☐	ChIP-seq
☒	☐	Flow cytometry
☒	☐	MRI-based neuroimaging

Human research participants

Policy information about studies involving human research participants

Population characteristics	See above.
Recruitment	Primary tumour tissue and matched blood samples were prospectively collected from patients undergoing curatively-intentioned surgery at University College London Hospital (UCLH).
Ethics oversight	UCLH Cancer Biobank (REC approval 15/YH/0311)

Note that full information on the approval of the study protocol must also be provided in the manuscript.
